# Study abroad programmes and student outcomes: Evidence from Erasmus^☆^

**DOI:** 10.1016/j.econedurev.2024.102510

**Published:** 2024-04

**Authors:** Silvia Granato, Enkelejda Havari, Gianluca Mazzarella, Sylke V. Schnepf

**Affiliations:** a European Commission, Joint Research Centre (JRC), Italy; b IESEG School of Management, Univ. Lille, CNRS, UMR 9221 - LEM - Lille Economie Management, F-59000 Lille, France; c Università degli Studi di Milano, Italy; d IZA Institute of Labour, Germany; e Global Labor Organization, Switzerland

**Keywords:** Study abroad, University, Erasmus, Regression discontinuity design

## Abstract

Exploiting admission thresholds for participating in Erasmus, the most popular higher education study abroad programme in Europe, we implement a regression discontinuity design and show that student mobility does not delay graduation and, in addition, has a positive and significant impact on the final graduation marks of undergraduate students. We find that Erasmus mobility improves graduation results for undergraduate students enrolled in scientific and technical fields and for those who apply in the first year of their studies, especially when enrolled in more demanding degree courses. Investigating plausible mechanisms, we find that the positive impact on performance at graduation is stronger for students who visit foreign universities of relatively lower quality compared to their home university. Finally, we do not find statistically significant effects of Erasmus mobility on postgraduate educational choices and labour market outcomes one year after graduation.

## 1 Introduction

Over the last thirty years, there has been a consistent increase in the number of university students worldwide who have pursued part of their education abroad. In the US, 350,000 students spent a period abroad for academic credit in 2018, with an average growth rate of 6% since 1989. By 2018, about one tenth of American college students had studied abroad during their undergraduate years.^1^ A similar trend is observed in the European Union (EU): in 2019, more than 400,000 EU graduates spent a period studying abroad, with approximately 60% receiving financial support from an EU programme. The most popular study abroad programme for university students in the EU is the Erasmus programme.^2^ It was established in 1987 to enable European university students to spend a period of time studying abroad in an EU member state, supported by mobility grants. Over time, the programme has widened its scope, with the current Erasmus+ 2021–2027 covering additional areas of education, including vocational training, school, adult education and sport, with a budget of €26.2 billion. In thirty years, the programme has promoted the mobility of approximately 4.5 million higher education students in more than 30 countries.

The overall stated objective of the Erasmus programme is ‘to support, through lifelong learning, the educational, professional and personal development of people in education, training, youth and sport, in Europe and beyond’. More specifically, it is expected that the policy generates positive effects on a wide spectrum of young people’s outcomes, including not only the development of soft skills and improved foreign language abilities but also improved learning performance,

enhanced employability and improved career prospects, as well as increased motivation for continuing education.^3^ This study focuses on the latter aspects and investigates the impact of participating in the Erasmus programme on student academic performance, as well as on post-graduation educational choices and labour market outcomes one year after graduation.

Studying abroad is associated with considerable time-intensive organisation tasks, the need to improve language skills and the need to adapt to a new learning environment characterised by different peer abilities, teaching approaches and student networks. These organisational tasks and changes in learning inputs may impact the academic performance of students, with implications for their future choices and their performance in further education, as well as in the labour market. To the best of our knowledge, this study is the first to investigate this topic and identify the causal effect of the programme on academic performance and post-graduation outcomes, as well as the mechanisms at play.

We use rich administrative data from the University of Bologna, the second-largest public university in Italy, and combine student academic records with information on applications to the Erasmus programme. Italy is among the EU countries with the highest number of Erasmus participants, alongside France, Germany and Spain. In 2019, Italian universities contributed over 14% of all credit mobile graduates participating in Erasmus (or other EU programmes) in the EU, with approximately 30,000 students.

The allocation mechanism of the Erasmus grants at the University of Bologna offers an ideal quasi-experimental setting that allows us to tackle the bias deriving from selection into the programme and pin down causal effects. More specifically, every year the available grants funding specific Erasmus programmes (a specific Erasmus programme is defined by the foreign university of destination and the length of the study period abroad) are assigned to students who achieve the highest positions in programme-specific rankings, based on a score calculated as a function of previous academic performance, language skills and the quality of the application. Students can turn down offers, and both those who are not awarded a grant and those who turn down awarded grants can reapply and participate in the programme later in their study career. We exploit this allocation mechanism using a fuzzy regression discontinuity design (RDD) and estimate the impact of participating in the Erasmus programme separately for samples of undergraduate and graduate students.

We find that spending a portion of university studies abroad does not have an impact on the probability of graduating on time for either group. Moreover, it has a positive effect on the final graduation mark of bachelor’s students only. The estimates show that the latter obtain a 2-point premium in their final grade, which is approximately a third of one standard deviation of the final grade in the estimation sample. This effect is mainly driven by an increase in student grade point averages (GPAs) before graduation, which reflects grades from all exams taken during the study course, including those completed abroad (net of extra points assigned at graduation for dissertation quality or other achievements). The results from the baseline analysis indicate that participating in Erasmus does not lead to detrimental effects on student academic performance.

We investigate heterogeneity in the impact across both student and study abroad programme characteristics in order to shed some light on the mechanisms at play. In particular, whether the impact on student academic outcomes reflects human capital gains/losses or other mechanisms has implications in terms of student career prospects and policy advice. A standard educational production function framework indicates that studying at a foreign university produces changes in learning outcomes through several potential channels. These include the change in learning inputs, encompassing the language of instruction, the quality of professors, peers, teaching and marking methods, as well as the amount and quality of resources offered to students at the host institution. Furthermore, other aspects of a study abroad experience potentially influence the process of learning. On the one hand, acquiring the organisational and adaptation skills necessary to live in a new environment requires a substantial investment of time and effort that may translate into poorer academic performance and a delay in the study career. On the other hand, the experience may be thought of as a positive input in the building of human capital and is potentially relevant for achieving better academic outcomes. Additional behavioural mechanisms may also be at play. For instance, students may put less effort into their studies while abroad, seeking adventure and excitement over academic career advancement, which – all other things being equal – may negatively impact academic results. More simply, the positive effect (or the absence of negative effects) of Erasmus participation on student academic outcomes might merely be a ‘mechanical’ result driven by students systematically obtaining higher grades in exams taken when studying abroad, because of generous grading at the host institution and/or generous grade conversion at home, as suggested by anecdotal evidence.

We find that the effect on the final graduation mark is remarkably stronger for graduates in science, technology, engineering and mathematics (STEM) and students who apply for the Erasmus programme earlier in their studies, suggesting that the observed impact might be related to the content of exams taken during the study period abroad. Most STEM subjects are based on maths, meaning that the disadvantage linked to language barriers is potentially mitigated. Moreover, first-year bachelor’s degree courses typically cover broad subjects that create the building blocks for a field of study and are usually worth more in terms of academic credits^4^; these often lead to ‘*cream-skimming*’, meaning that only relatively higher quality students are able to pass them and progress in their careers, especially in typically more challenging degree courses, such as STEM ones. We find that the Erasmus ‘advantage’ is concentrated among students applying for mobility programmes early on and enrolled in more demanding degree courses – courses in which students tend to accumulate fewer ECTS in the first year – indicating that higher grades during Erasmus might allow some students to overcome first-year cream-skimming.

In further investigating potential mechanisms, we find that the positive effect on the final graduation mark appears to be driven by programmes in host institutions of relatively lower quality – and thus arguably with a relatively lower quality of learning inputs – and, in particular, when the duration of the period abroad is longer.

Taken together, our findings seem to point to the fact that the observed effects on academic performance can be attributed to a direct impact on student achievement *during* the study period abroad, rather than a more general impact on the learning performance of students, which would be reflected in student performance *after* the experience abroad and could signal human capital accumulation. We provide additional descriptive evidence pointing in the same direction, indicating that the exam marks of Erasmus students are higher only while abroad – and in particular in host institutions of lower relative quality – and not after returning home.

Importantly, the impact of international student mobility (ISM) on students’ performance at university has implications for their paths after exiting education. Both human capital theory and signalling theory predict that graduates’ academic performance contributes to their employability and earnings (see Arteaga, 2018, Naylor et al., 2002 for example, for a discussion). Various studies provide evidence on the causal relationship linking final university grades and time to graduation to graduate employment opportunities and earnings (see Aina and Casalone, 2020, Aina and Pastore, 2020, Feng and Graetz, 2017, Freier et al., 2015, Naylor et al., 2015). Furthermore, graduation marks and time to graduation realistically influence graduate decisions to pursue further education, with similar implications for future career prospects. We merge student administrative data with survey data on student choices and outcomes one year after graduation and investigate the potential impact of study abroad on the probability of continuing studies and of being employed. We do not find significant effects on these outcomes, although our analysis is likely hampered by the small sample size.

Our paper contributes to the existing literature on the effects of international student mobility by offering novel robust causal evidence on various student outcomes — notably academic performance, which had remained largely unexplored. The previous literature has mostly relied on survey data measured after a study abroad experience and has estimated the effect of international student mobility (through Erasmus or other programmes) on longer-term outcomes, mainly relying either on propensity score matching methods or on instrumental variable approaches.^5^ Most studies have focused on future mobility and labour market performance and have found positive effects on the probability of living and/or working abroad (Oosterbeek and Webbink, 2011, Parey and Waldinger, 2011, Rodrigues, 2013) and on employment status and earnings (Di Pietro, 2015, Messer and Wolter, 2006). Other studies have documented positive impacts on the development of specific skills, including foreign language proficiency (Sorrenti, 2017) and intercultural competence (Salisbury et al., 2013). The only evidence on academic performance is provided by Cullinan et al. (2022), who find no independent association between study abroad and Irish students’ GPAs after returning to their home university. Thanks to the availability of administrative data on individual study careers containing information on both student applications for study abroad and mobility and on student outcomes measured before their exit from education, we are able to investigate the impact of the most popular EU study abroad programme from a different perspective, i.e. exploiting the discontinuity in Erasmus participation probability at the cutoff values of a student ability measure and looking at previously overlooked relevant outcomes. Furthermore, our research design is novel in that it makes use of very recent methodological advancements developed in other contexts (Abdulkadiroglu et al., 2022, Abdulkadiroglu et al., 2017, Fort et al., 2022, Fort et al., 2020) regarding how to generalise regression discontinuity designs to allow for multiple cutoffs and multiple running variables. To the best of our knowledge, our study is the first application of this method to the investigation of ISM impacts.

The remainder of the paper is organised as follows. Section 2 describes the selection process for participating in the Erasmus programme at the University of Bologna. Section 3 discusses how the regression discontinuity design is implemented to estimate the causal impact of participating in the Erasmus programme. Section 4 describes the data and presents evidence of the validity of our empirical design. Section 5 presents the main results and the evidence from the heterogeneity analysis, while Section 6 further discusses potential mechanisms behind the findings. Section 7 offers some concluding remarks.

## 2 Institutional background

The University of Bologna is the oldest in Europe and one of the largest public universities in Italy, attracting approximately 5% of all students enrolled in higher education in Italy every year.^6^ Among Italian universities, the University of Bologna also has one of the strongest traditions of participation in the Erasmus programme: 11.2% of all students graduating in 2019 spent a period studying abroad with an Erasmus scholarship (or through another EU programme), while this figure is 9.6%, on average, across the majority of all other Italian higher education institutions.^7^

**Fig. 1:**
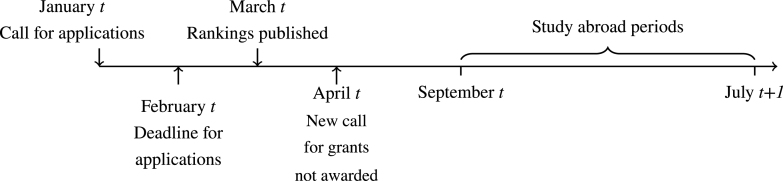
Timeline of the application process.

Fig. 1 shows the timeline of the Erasmus application process at the University of Bologna in a given academic year.

At the beginning of the calendar year (January of year t), the University of Bologna publishes a call for applications to take part in the Erasmus programme in the following academic year (i.e. to spend a period of study abroad of varying duration between September of calendar year t and July of calendar year t+1). Each department has agreements with departments at other universities that are part of the Erasmus programme. The number of grants available for a study abroad programme at a given host institution and of a given length (which we refer to as a *specific Erasmus programme*) is defined within agreements between the departments of the University of Bologna and those of the host institutions. Until 2018, in the January call each student could submit a maximum of two applications for the Erasmus opportunities available within her department. To illustrate this using an example, consider that in a given academic year the Department of Economics at the University of Bologna has Erasmus programme agreements with X universities in Y different countries (with X≥Y), each for a bilaterally agreed-upon length time; thus, in the January call of that academic year, a student from the Department of Economics could submit two Erasmus applications to two different universities (in the same or different countries). The student could thus apply for two *specific Erasmus programmes* and, if eligible, would participate in two different rankings (rankings are programme-year-specific). If the student qualifies for the Erasmus grant for both universities, at least one offer has to be turned down.

To be eligible for the Erasmus programme and receive the mobility grant, a student must have at least elementary proficiency in the language of instruction at the host university (A2 level, as per the Common European Framework of Reference for Languages), as well as a study plan for her time abroad. After applying, students receive a score from 0 to 100 based on their academic history and the quality of their application. Up to 60 points are assigned based on the average grade and the number of academic credits (measured according to the European Credit Transfer System) accumulated up to the year and month of application (generally February t)^8^; the remaining 40 points are assigned by the university staff member managing the programme based on the quality of the applicant’s study project, her motivation letter and her language proficiency.

Students applying for a specific programme are ranked based on this score, and the available grants are assigned to the highest-ranked eligible candidates. After the programme rankings are published, students who are awarded grants have approximately one week to decide whether to accept or turn down the offer. Subsequently, based on these decisions, the turned-down grants are reassigned to the next eligible student in the programme’s ranking, continuing until the last eligible student. If there are still vacant places after this process, a second round of applications is launched and the procedure is repeated. However, in this second round students can only submit one application. Students can also decide to renounce the grant at a later stage, after having initially accepted. Furthermore, students who are awarded and accept the grant can be rejected by the host institution due to not fulfilling specific requirements (e.g. deadlines, etc.). In such cases, the grants of renouncing/rejected students are not reallocated.

Students can apply in multiple years throughout their studies and can receive more than one Erasmus mobility grant even within the same study cycle,^9^ provided that the cumulative duration of the period abroad does not exceed 12 months (or 24 months for single-cycle degrees).

## 3 Empirical strategy

The main identification issue that arises in trying to estimate the causal effects of participating in the Erasmus programme is that students are not randomly assigned to the ‘treatment’ and, thus, are likely to have observable as well as unobservable characteristics potentially correlated both with the probability of participating in the Erasmus programme and with graduation and post-graduation outcomes (e.g. ability, motivation and open-mindedness). To tackle this selection issue, we exploit the allocation mechanism of the Erasmus mobility grants at the University of Bologna in a fuzzy regression discontinuity design: under certain assumptions, close to each programme-specific qualifying cutoff the grant assignment can be considered *as good as random*.

Our starting population is composed of students who applied for an Erasmus mobility grant at least once during their higher education career. We consider an individual as ‘treated’ if she ever participated in the Erasmus programme in her study career. We observe the yearly outcomes of the mobility grant assignment process, i.e. the final applicant ranking for each specific programme, including students’ decisions to accept or turn down the offer(s) and their final status as Erasmus participants.

Within each of these programme-year-specific rankings, we identify the qualifying cutoff score as the score of the last student who is offered one of those grants, regardless of whether she participated in Erasmus (i.e. including students who turned down/renounced the offer or were rejected by the host institution). It is worth noting that in each programme-year-specific ranking, because of students turning down offers and turned-down grants being reallocated, the position in the ranking of the last student who is offered a grant can be higher than the number of available grants. On the other hand, the student just below the threshold is the first of those to not receive a grant offer. The running variable for each student in the ranking is constructed as her score normalised to the cutoff score, so that it has a value of zero for the last student who is offered the scholarship and takes a positive (negative) value for those ranked higher (lower).^10^

Students can participate in several calls for applications in different academic years, as well as apply for different specific Erasmus programmes within the same academic year (with a maximum of 3, i.e. 2 in the January call and 1 in the spring call). Therefore, each student will have as many running variables as programme-year-specific rankings in which she participates. In order to deal with this feature of the allocation mechanism, similar to Fort et al. (2020), for each student we first focus on the January call for applications in the first academic year of participation; one can indeed consider that every subsequent participation in a call is partly affected by the outcome of the first. Second, for students who apply to two different specific programmes within the January call of the first academic year of participation, we take the running variable with the maximum value, which proxies the student’s effective probability of receiving *at least* one grant offer. These students’ order preferences for their applications (if any) are not made explicit at the moment of the application and do not ex-ante affect the probability of receiving a grant offer. Thus, we argue that the cutoff of the programme-year-specific ranking in which the applicant’s running variable has the maximum value is the only ‘relevant’ cutoff and that we can eliminate selection bias by comparing individuals whose highest scores are just above and just below this cutoff.

In our setting, being above or below the cutoff does not exactly determine the treatment status. More specifically, non-compliance is given by non-treated students at or above the cutoff, i.e. those who have been offered at least one mobility grant in the first academic year of application in their study career but turned down (or renounced/were rejected for) all offered grants, even at later stages of their study career, and treated students below the cutoff, i.e. those who were not offered any grant in the first academic year of application but participated in an Erasmus programme at a later stage in their study career.

We estimate the effect of participating in the Erasmus programme on student academic and post-graduation outcomes via an instrumental variable approach. Our equation of interest is

(1) $$Y_{ir}=\beta_{1}T_{i}+\beta_{2}f\left(x_{ir}\right)+\mu_{r}+\epsilon_{ir},$$

where $Y_{ir}$ is the outcome of student i who has her maximum normalised score – among her applications in the January call of her first year of application – in the programme-specific ranking r. $T_{ir}$ is the treatment variable, which takes the value of 1 if the student has ever studied abroad through an Erasmus programme in her study career. $x_{ir}$ is the running variable measured in programme-year-specific ranking r, i.e. the maximum of the student’s normalised scores among applications in the January call of her first year of application, and f(⋅) is a polynomial in the running variable. Given that each programme-year-specific ranking has its own cutoff score, normalising the running variable according to the score of the last student offered a mobility grant implies that there will be a bulk of observations with the value of the running variable equal to zero. As shown by Fort et al. (2022), in this type of setting the bias in the estimated regression discontinuity design coefficient is eliminated by including programme-year-specific ranking fixed effects $\mu_{r}$. $\epsilon_{ir}$ is an individual specific error term.

The corresponding first-stage equation is

(2) $$T_{ir}=\alpha_{1}Z_{ir}+\alpha_{2}f\left(x_{ir}\right)+\zeta_{r}+\eta_{ir},$$

where $Z_{ir}$ is a dichotomous indicator for having a (normalised) score equal to or above the (zero) cutoff, i.e. Z=1(x≥0). $\zeta_{r}$ are programme-year-specific ranking fixed effects, and $\eta_{ir}$ is an individual error term. We cluster standard errors at the programme-year-specific ranking level. The estimation of the causal parameter of interest relies on the assumption of monotonicity of the treatment in the instrument being satisfied, i.e. absence of defiers. In our context, this implies assuming that there are neither applicants who would participate in the Erasmus programme during their study career only if they were not offered an Erasmus grant in their first year of application, nor applicants who would not participate in the Erasmus programme only if they were offered an Erasmus grant in their first year of application. Under this assumption, our coefficient of interest, $\beta_{1}$, measures the *local average treatment effect for compliers at the cutoff*. Appendix A.1 contains a more detailed discussion of non-compliance in our setting. It includes an investigation of the validity of the monotonicity assumption and descriptive evidence in favour of it. In particular, looking at student treatment status, distinguishing participating in Erasmus in the first year of application or subsequent years conditional on grant assignment status in the first year of application (see Table A4), it emerges that the majority of students who are offered mobility grant(s) the first year of application accept the offer and participate in Erasmus at that time, and the majority of those who are not offered the grant in the first year of application never participate in Erasmus during their study career. We interpret this evidence as suggestive of the absence of defiers. Moreover, we show that under this assumption, our design allows estimating the treatment effect on a specific sub-population of compliers, i.e. students who participate in the Erasmus programme as an outcome of their first application year and would not have participated in Erasmus in their study career had they not been offered the grant in their first application year. Finally, it is important to note that our design allows estimating the treatment effect for compliers at the threshold point, i.e. the qualifying cutoff of the application score. The latter is a function of student skills (language and past academic performance) and application quality and, thus, it is positively correlated with student *quality*.^11^ This implies that we are not able to identify the treatment effect for the *best* (well above the threshold) or the *worst* (well below the threshold) students.

Our research design is close in spirit to the methodology developed in Abdulkadiroglu et al. (2022) in the context of centralised school assignment. We discuss the similarities between the two approaches in Appendix A.2 and provide an additional robustness check inspired by their approach.

## 4 Data

We use data on applications submitted for all Erasmus grants funding a period of study abroad available to students at the University of Bologna between the 2013/2014 and 2018/2019 academic years.^12^ A rich set of information is available for each specific programme funded, including the department of the home university managing the agreement, the number of available grants, the length of the period abroad according to the initial agreement (which can be reduced or extended upon specific request from the student once she is abroad and with the approval of both the home and foreign university), the country of destination and the name and location of the host institution. These data were made available by the office responsible for the management of study abroad programmes at the University of Bologna.

Overall, in the period considered there were approximately 36,500 applications to 10,127 specific Erasmus programmes. Table B2 in Appendix B reports some descriptive statistics on this sample of applications. Over the entire period considered, the average number of Erasmus grants available for each specific programme was 2.3, and each programme received, on average, 3.6 applications, indicating that there is competition to obtain the available grants. The majority of programmes (slightly more than half) fund periods of study abroad of 5 to 6 months or 9 to 10 months (approximately 40%). Overall, the University of Bologna established agreements for students to study abroad with more than 700 universities in 47 countries; the largest share of agreements are with universities in Spain (25%), followed by France and Germany (15% and 11%, respectively).

We match data on applications with administrative records on individual demographic and study career information (course of study, number of exams and ECTS credits accumulated and average exam grades by calendar year, as well as the date and grade of graduation for students who completed their study cycle) for students who enrolled in a study career at the University of Bologna from the 2007/2008 academic year onwards. These data were made available by the statistical office of the University of Bologna.^13^ We focus on bachelor’s and master’s students whose study careers as of the end of 2019 (when the data were extracted) should have already been concluded according to the legal duration of their study course. We then focus on students who have graduated, excluding those who dropped out (1.6% of the sample of students whose study careers should have been concluded by 2019) and those who are still enrolled due to a delay in completion (another 16.4%).

To ensure that through our empirical strategy we are identifying and comparing students who *just* received or *just* did not receive the Erasmus grant, we first eliminate students who, in their first year of application, participated in at least one specific programme that has a number of applicants lower than the number of available grants (36% of the sample). In fact, for programme-year-specific rankings in which there are not enough eligible students applying and not all of the grants available are offered to students, it is not possible to identify the last offered and the cutoff score; consequently, for students participating in at least one of these rankings, the maximum value of the running variable is not defined. Second, we further eliminate from the sample another 11% of students who have their first-application-year maximum normalised score in a ranking in which the number of applicants is equal to the number of available grants.

The final sample comprises 3,912 bachelor’s students and 2,396 master’s students. Table 1 displays summary statistics for the two final samples separately for students who never participated in Erasmus during their study career (‘No Erasmus’ columns) and students who participated in the Erasmus programme at least once in their study career (‘Erasmus’ columns).^14^ Approximately 59% of bachelor’s students and 57% of master’s students participated in the Erasmus programme at least once during their study career.^15^ Females and students in the field of education, arts and humanities appear to be slightly over-represented among Erasmus participants, in particular in the bachelor’s student sample. On average, bachelor’s and master’s students who participated in an Erasmus programme at least once during their study career had accumulated a lower number of passed exams and ECTS credits when they first applied. This is explained by the fact that relative to non-treated students they are more likely to have applied for the first time earlier in their university studies.

In the bottom part of the table, we report summary statistics for the four main outcomes of interest measuring success in student university careers, namely the probability of graduating on time, time to graduation, the final graduation mark and the probability of graduating with *distinction*. In the Italian higher education system, each student has to pass exams for a given number of ECTS credits every year in order to enrol in the next year of the study career and be on time. The first outcome measures the probability of being on time when graduating. The time to graduation is the number of months between October of the academic year of enrolment in the first year of the study career (the conventional month of first enrolment) and the month of graduation. Final graduation marks range between 66 and 110, and particularly high-achieving students can obtain their qualification *cum laude*, referred to here as *distinction*. The evidence in Table 1 indicates that for the bachelor sample only, Erasmus participants take less time to graduate, as indicated by both a higher probability of graduating on time and a lower time to graduation in months. In both samples, Erasmus participants obtain a higher final graduation mark and have a higher probability of graduating with distinction, on average.

**Table 1:** Descriptive statistics by treatment status.

Variables:	Degree level							
	Bachelor				Master			
	Mean	S.d.	Mean	S.d.	Mean	S.d.	Mean	S.d.
	No Erasmus		Erasmus		No Erasmus		Erasmus	
**Student characteristics**								
Female	0.57	(0.5)	0.65	(0.48)	0.51	(0.5)	0.55	(0.5)
Moved from other region	0.5	(0.5)	0.57	(0.5)	0.65	(0.48)	0.65	(0.48)
Foreign-born	0.07	(0.26)	0.05	(0.22)	0.14	(0.35)	0.1	(0.3)
*Field of study:*								
Education-Arts-Humanities	0.23	(0.42)	0.35	(0.48)	0.16	(0.37)	0.23	(0.42)
Social sciences	0.37	(0.48)	0.41	(0.49)	0.33	(0.47)	0.33	(0.47)
Business-Admin-Law	0.19	(0.39)	0.11	(0.31)	0.2	(0.4)	0.15	(0.36)
STEM	0.14	(0.35)	0.09	(0.28)	0.29	(0.45)	0.26	(0.44)
Health & Agricultural sciences	0.07	(0.26)	0.05	(0.22)	0.03	(0.16)	0.03	(0.17)
**Applications**								
Total no. of applications:	1.88	(0.95)	2.24	(0.97)	1.77	(0.75)	1.91	(0.68)
No. of applications by ac.year:	1.66	(0.65)	1.88	(0.57)	1.71	(0.66)	1.84	(0.57)
*Career year of first application (bachelor):*								
First	0.25	(0.43)	0.35	(0.48)	–	–	–	–
Second	0.6	(0.49)	0.62	(0.48)	–	–	–	–
Third and beyond	0.15	(0.35)	0.03	(0.16)	–	–	–	–
*Career year of first application (master):*								
First (or 3rd bachelor)	–	–	–	–	0.93	(0.25)	0.95	(0.21)
Second and beyond	–	–	–	–	0.07	(0.25)	0.05	(0.21)
*Academic performance at application:*								
No. of exams at 1st application	5.8	(4.32)	4.44	(3.32)	0.67	(1.42)	0.56	(1.44)
No. of ECTS at 1st application	49.38	(35.33)	38.96	(28.62)	5.19	(11)	4.17	(10.38)
**Outcomes at graduation**								
Graduated on time	0.85	(0.35)	0.92	(0.27)	0.8	(0.4)	0.81	(0.39)
Time to graduation (months)	38.26	(7.74)	37.01	(6.35)	29.12	(6.52)	29.01	(5.49)
Final graduation grade	100.38	(8.06)	104.29	(6.27)	106.32	(5.19)	107.63	(3.88)
Prob. of distinction	0.15	(0.36)	0.25	(0.44)	0.36	(0.48)	0.47	(0.5)

Observations	1601		2311		1020		1376	

*Notes:* The table reports summary statistics for the final samples of bachelor’s and master’s students, separately for students who never participated in the Erasmus programme during their study career (‘No Erasmus’ columns) and students who participated in the Erasmus programme at least once in their study career (‘Erasmus’ columns). The final sample is made up of students who enrolled in the first year of a study career at the University of Bologna from the 2007/2008 academic year onwards and applied for the Erasmus programme for a study abroad period between the 2013/2014 and 2018/2019 academic years, and who had graduated by the end of 2019 (when data were extracted).

### 4.1 First stage and tests of the identifying assumptions

**Fig. 2:**
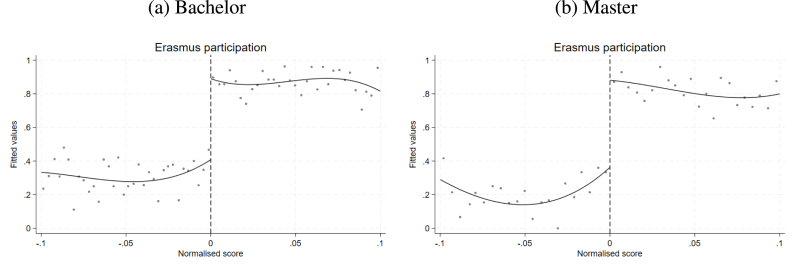
First-stage plot. Notes: The figure plots averages within bins of the running variable on the x-axis and a dummy for being ‘treated’ on the y-axis. The running variable is defined as the maximum of the normalised distance to the cutoff scores from different applications in the January call of the first year of participation in a call for applications. The cutoff score is the score of the last student offered the scholarship in each programme-specific ranking. The dummy for being treated is constructed as being 1 for any student who has participated in the Erasmus programme at least once during her study career. The number of bins is calculated with the mimicking variance evenly spaced method using spacings estimators. The relationship is fitted with a polynomial of order three.

Fig. 2 is a graphical representation of the first stage, i.e. the relationship between the running variable and the treatment variable. We use a cubic specification and do not condition on other covariates.^16^ The figure shows a clear jump in the probability of participating in the Erasmus programme due to the grant assignment mechanism, both for the sub-sample of bachelor’s students – panel (a) – and the sub-sample of master’s students – panel (b). Table B4 in Appendix B reports the results of the estimation of the first-stage equation performed on the samples obtained imposing a bandwidth of 0.1 around the cutoff.^17^ The first-stage coefficient is positive and significant at the 1% level; it indicates that having a score at or above the cutoff increases the probability of participating in the Erasmus programme by approximately 52 percentage points for bachelor’s students and 61 percentage points for master’s students.

The identifying assumption of our design is that individuals do not have precise control over the score received (no manipulation of the running variable). Hence, being the last student offered the mobility grant or the first excluded can be considered ‘as good as random’. This assumption ensures that, on average, treated and control units around the cutoff have similar observable and unobservable characteristics. The bulk of observations with a value of the running variable equal to zero, generated by normalising the running variable according to the programme-year-specific ranking cutoff score, will produce a discontinuity in the distribution of the running variable, which translates into a failure in the standard test of manipulation of the running variable (McCrary, 2008), as shown in the left panels of Figure B1 in Appendix B. Including programme-year-specific ranking fixed effects in the analysis, and thus exploiting only within-ranking variability, allows us to address this issue. The right-hand panels of Figure B1 illustrate that the distribution of the residuals of the running variable after the inclusion of ranking fixed effects is not discontinuous at the cutoff.

In addition, we run a series of estimations to check that there is no jump at the cutoff for pre-treatment variables of interest. Specifically, we look at academic performance before the application, measured by the number of exams and ECTS credits students accumulated prior to their first application, which we are able to observe until the end of the calendar year prior to the calendar year of application. Moreover, we investigate potential discontinuities in both the gender and country of birth of applicants – through indicators for being female and for being born outside of Italy – and in a potential measure of attitudes towards mobility, namely the probability of having moved from another region to study at the University of Bologna (similar to Sorrenti, 2017). Table 2 reports the results from the estimation of both a reduced-form equation (RF, columns 1 and 3) and an instrumental variable approach (IV, columns 2 and 4) where the outcome variables are those described above, showing that the selected pre-treatment variables are balanced around the cutoff.

**Table 2:** Smoothness of pre-determined covariates.

	(a) Bachelor		(b) Master	
	(1)	(2)	(3)	(4)
	RF	IV	RF	IV
*Dependent variables:*				
Female	−0.02	−0.038	−0.04	−0.066
	(0.045)	(0.086)	(0.085)	(0.14)
Foreign-born	−0.024	−0.047	−0.006	−0.01
	(0.024)	(0.046)	(0.054)	(0.089)
Moved from other region	−0.033	−0.064	0.087	0.143
	(0.047)	(0.09)	(0.075)	(0.124)
No. of ECTS at application	0.689	1.323	−1.508	−2.48
	(2.265)	(4.367)	(1.235)	(2.017)
No. of exams at application	0.137	0.264	−0.206	−0.338
	(0.26)	(0.502)	(0.175)	(0.285)
Observations	1852	1852	899	899

*** p < 0.01, ** p < 0.05, * p < 0.1

*Notes:* The table reports the coefficients and standard errors from the estimation of a reduced-form equation (columns 1 and 3 for the samples of bachelor’s and master’s students, respectively) and an IV regression (columns 2 and 4 for the samples of bachelor’s and master’s students, respectively) with a running variable within a bandwidth of 0.1 for five pre-treatment variables: an indicator for being female; an indicator for being born outside of Italy; an indicator for having moved from another Italian region to study at the University of Bologna; the number of ECTS credits accumulated at the end of the calendar year preceding the calendar year of the application; the number of exams passed at the end of the calendar year preceding the calendar year of the application. All specifications include a polynomial of the running variable of order 1 and are estimated using triangular kernels. Errors are clustered at the programme-year-specific ranking level. Robust standard errors are in parentheses.

## 5 Results

### 5.1 Baseline results

In this section, we report and discuss the main results for our outcomes of interest, namely, the two measures of time to graduation – the probability of graduating on time and the number of months to graduation – and the two measures of performance at graduation – final graduation mark and the probability of graduating with distinction. It is worth clarifying that in the Italian university system, a student’s exit grade is made up of different components. First, the average grade of all exams, including those passed abroad during Erasmus, determines the base graduation mark. Exam grades obtained by students in a foreign institution but within the Erasmus programme are converted into grades at the home institution according to grading scales introduced by the European Commission (EC) in 2009 that make grades comparable across European universities. Grading scales are statistical distribution tables of the grades awarded in a programme or field of study at a given institution, which allow for comparison with the statistical distribution of grades in a parallel reference group at another institution (see European Commission, Directorate General Education And Culture, 2015 pp. 39–40).^18^ Second, at graduation additional points can be awarded based on the quality of the final thesis. Finally, each degree course can establish its own rule regarding whether (and how) to attribute additional points to students graduating on time (or earlier) or/and participating in international mobility programmes. We choose to focus on the final graduation mark resulting from all of the different components because it is the official final exit grade that will appear on students’ CVs and will matter for their future decisions, thus it is more salient. When presenting the results, we provide evidence and discuss the role of the different components in driving the observed effects.

Table 3 reports the results regarding the four outcomes of interest for bachelor’s students (panel (a)) and master’s students (panel (b)). A visual inspection of the relationships between the running variable and the outcomes of interest (as provided in Fig. 2 for the probability of Erasmus participation) is provided in Figure B2 in Appendix B. All estimations are performed on the samples obtained imposing a bandwidth of 0.1 around the cutoff. The table displays both the results of the estimation of a reduced-form (RF) equation with polynomials in the running variable of order one (columns 1, 3, 5 and 7 in both panels) and the results of an instrumental variable (IV) regression (columns 2, 4, 6 and 8 in both panels). Looking at the two measures of time to graduation (columns 1 to 4 of each panel), no significant effect emerges across the different specifications and models for either sub-sample. We test the robustness of these results to alternative ways of measuring time to graduation, in particular investigating the effect on the probability of graduating within different, increasing, intervals of time. The results are reported in Table B5 in Appendix B and confirm that participation in the Erasmus programme does not significantly impact time to graduation. The evidence that participation in the Erasmus programme does not delay time to graduation is particularly relevant in the Italian context, where higher education is subsidised, late graduation rates are some of the highest among OECD countries (Sorrenti, 2017) and late graduation implies significant penalties in terms of employment probability, earnings (Aina & Casalone, 2020) and job-match quality (Aina & Pastore, 2020).^19^

When examining the impact of Erasmus participation on the two outcomes measuring students’ exit grades, a significant positive effect is observed on the final graduation mark for bachelor’s students only (columns 5 and 6 in the top panel). In particular, Erasmus participation causes an increase of up to 2 points in the final graduation mark, which is significant at the 5% level. The magnitude is one third of one standard deviation of the final grade in the estimation sample. All results for the four main outcomes of interest are robust across different bandwidths, as shown in Figure B3 in Appendix B.

**Table 3:** Main results.

(a) Bachelor sample								
*Dependent variables:*	Prob. of graduating on time		Time to grad. (months)		Final graduation mark		Prob. of distinction	
	(1)	(2)	(3)	(4)	(5)	(6)	(7)	(8)
	RF	IV	RF	IV	RF	IV	RF	IV
Above cutoff score ($Z_{ir}$)	0.011		−0.207		1.094**		0.059	
	(0.017)		(0.368)		(0.510)		(0.038)	
Erasmus participation ($T_{i}$)		0.021		−0.397		2.100**		0.114
		(0.032)		(0.706)		(0.954)		(0.073)
								
Observations	1852	1852	1852	1852	1852	1852	1852	1852
R-squared	0.550		0.602		0.601		0.513	

(b) Master sample								
*Dependent variables:*	Prob. of graduating on time		Time to grad. (months)		Final graduation mark		Prob. of distinction	
	(1)	(2)	(3)	(4)	(5)	(6)	(7)	(8)
	RF	IV	RF	IV	RF	IV	RF	IV
Above cutoff score ($Z_{ir}$)	0.003		−0.017		0.709		0.044	
	(0.042)		(0.592)		(0.654)		(0.075)	
Erasmus participation ($T_{i}$)		0.005		−0.028		1.166		0.072
		(0.069)		(0.973)		(1.082)		(0.123)
								
Observations	899	899	899	899	899	899	899	899
R-squared	0.544		0.582		0.487		0.499	

*** p < 0.01, ** p < 0.05, * p < 0.1

*Notes:* The table reports the results of the estimation of a reduced-form equation (columns 1, 3, 5 and 7 in each panel) and an IV regression (columns 2, 4, 6 and 8 in each panel) for samples of bachelor’s – panel (a) – and master’s – panel (b) – students, with a running variable within a bandwidth of 0.1, for the four main outcomes: the probability of graduating without delay; the time to graduation measured in months; the final graduation mark (which ranges between 66 and 110); and a dummy for graduating with distinction. All specifications include a polynomial of the running variable of order 1 and are estimated using triangular kernels. All specifications include programme-year-specific ranking fixed effects (600 for the bachelor sample and 318 for the master sample). Errors are clustered at the programme-year-specific ranking level. Robust standard errors are in parentheses.

As mentioned above, some degree courses may establish rules that attribute additional points at graduation to students who graduate on time or earlier and/or students who have participated in the Erasmus programme. Precise information on this system at the degree-course level has never been collected systematically and is difficult to retrieve. Given that no significant impact is found on the time to graduation, we can exclude that the positive effect on graduation grade derives from the points premium attributed to reduced time to graduation. Furthermore, we can investigate whether participation in Erasmus has an impact on the base graduation mark, i.e. average exam grades calculated on all passed exams until the end of the study career, which are clean of any effect on marks attributed at graduation. Table B6 displays the results and shows a positive effect on average grades before graduation for bachelor’s students (and no effect for master’s students). Furthermore, the magnitude of the coefficients is similar to that estimated for the final graduation mark (32% of one standard deviation of average grades in the estimation sample). Therefore, we can also exclude that the impact on the final graduation mark is driven by the university policy of attributing a premium to Erasmus participants at graduation.

To sum up, we find that participating in the Erasmus programme does not delay or speed up graduation for bachelor’s students and has a positive effect on their graduation grade, mainly due to better performance in exams undertaken during the study career. The exam grades of Erasmus participants include grades of exams taken abroad, which, as mentioned above, are converted into grades at the home institution according to statistical tables. In principle, the grade distribution table developed for a specific reference group (single/group of degree courses) allows for a single grade obtained for a course unit abroad to be positioned in the context of the home university’s grading table, thus leading to an *objective* conversion of grades. Despite this, anecdotal evidence indicates that not all Erasmus universities release reference-group-specific statistical tables. Some only make available country-level aggregated statistics on grade distribution across 5 macro-groups of grades, potentially leading to *generous* grade conversion at home. Unfortunately, this information is not available at the level of individual institutions and, thus, we cannot directly test for this channel. In the following sections, we exploit the information available in our data to investigate potential mechanisms of the observed effect for bachelor’s students.

Overall, for master’s students the results suggest that Erasmus mobility does not have any impact on academic performance. These students have specific characteristics that may play a role in whether and how a study experience abroad might affect their academic outcomes. On the one hand, they are not undertaking their first degree, and being both more senior and older might make them less sensitive to the shock of moving abroad and to a change in learning inputs. On the other hand, they may have already participated in study abroad experiences in their previous degree, with similar implications. As a consequence, the rest of the paper focuses on explanations for the observed effects on the sample of bachelor’s students. For completeness, the same analyses are performed on the sample of master’s students and are reported in Appendix B, and the absence of significant evidence from these is discussed in the text where relevant.

### 5.2 Heterogeneous effects

We explore the heterogeneity in the effects for bachelor’s students across characteristics of interest, namely, the field of study as well as the timing of a student’s first application to the Erasmus programme within their study career. The latter can be interpreted as a proxy for their motivation to participate in the Erasmus programme and should be a predictor of the effective timing of the study abroad experience during the study career.

We estimate heterogeneous effects by instrumenting the interactions of the endogenous treatment status and the characteristic of interest with the interactions of the predictions of the first-stage regression and the same characteristic and report these IV estimates.

Panel (a) of Table 4 shows that the positive effect on the final graduation mark (column 3) for bachelor’s students is significant and larger in magnitude for students in the science, technology, engineering and mathematics (STEM) fields only. For STEM students, participation in the Erasmus programme causes an increase in their final graduation mark of approximately 6 points, which is almost one standard deviation of the average final grade in this sub-sample, and the coefficient is significant at the 1% confidence level. This effect is not driven by reduced time to graduation, as indicated by columns 1 and 2 in the same panel. The most obvious feature that differentiates the scientific and technical fields from the other subject areas is the maths content of exams. This attribute has potential implications for performance in exams taken abroad, as part of the disadvantage associated with studying and taking exams in a foreign language may be mitigated. Thus, we might think that for non-scientific/technical subjects, a positive effect on exam performance is attenuated by the language barriers, while this is not the case for STEM subjects.

When looking at the timing of the first application (panel (b) of Table 4), it emerges that only bachelor’s students who apply for Erasmus early in their study career, i.e. when enrolled in their first year, benefit from participating in the programme, as demonstrated by the negative and significant coefficient for time to graduation (column 2) and the positive and significant effects on both the final graduation mark and the probability of graduation with distinction (columns 3 and 4, respectively). For these students, participating in the Erasmus programme reduces time to graduation by approximately 2 months (53% of one standard deviation of this outcome for this sub-group in the estimation sample) and increases the final graduation mark by 4.6 points and the probability of graduating with distinction by 21 percentage points (85% and 49% of one standard deviation of these outcomes for this sub-group in the estimation sample, respectively). The results also show a negative effect on time to graduation for students first applying for Erasmus during or beyond the last year of their study career (column 2). These students spend their period abroad when they are already delayed in their career (i.e. from the 4th year onwards); for them, our finding indicates that the study period abroad delays their study career even further. On the one hand, independently of the effective timing of the experience abroad, students who apply for the Erasmus programme earlier could be those who are most motivated to participate in the programme and thus put more effort into their studies while abroad. On the other hand, the year of first application should be a good predictor of the effective timing of the period abroad, which could influence both the learning process and the time management of one’s study career. One potentially relevant aspect is the type and content of exams, which vary considerably across different years of a study career. Typically, in the first and (somewhat) in the second year, bachelor’s degrees offer courses on broad subjects that create the building blocks for a field of study, with these usually being mandatory and potentially of larger value in terms of ECTS. Courses on narrower topics are offered in the second part of a course cycle. Thus, first-year courses often lead to ‘*cream-skimming*’, meaning that only relatively higher quality students are able to pass them and progress in their careers, especially in typically more challenging degree courses such as those in STEM.

**Table 4:** Heterogeneity of effects across students characteristics – Bachelor sample – IV estimates.

(a) Differential effects by field of study				
*Dependent variables:*	Grad. on time	Time to grad.	Final mark	Distinction
	(1)	(2)	(3)	(4)
$T_{i}\;\times$ Arts & Humanities	0.029	−0.159	0.368	0.085
	(0.062)	(1.222)	(1.422)	(0.127)
$T_{i}\;\times$ Social sciences, business & law	0.021	−0.138	2.069*	0.141*
	(0.037)	(0.733)	(1.070)	(0.078)
$T_{i}\;\times$ Science, engineering & maths	0.015	−2.160	6.017***	−0.025
	(0.066)	(1.621)	(1.739)	(0.118)
$T_{i}\;\times$ Health & agricultural sciences	0.004	−1.087	2.322	0.193
	(0.028)	(1.506)	(1.885)	(0.177)
				
Observations	1852	1852	1852	1852

(b) Differential effects by study-career year of first application				
*Dependent variables:*	Grad. on time	Time to grad.	Final mark	Distinction
	(1)	(2)	(3)	(4)
$T_{i}\;\times$ 1st year	0.044	−2.107**	4.607***	0.209**
	(0.035)	(0.860)	(1.064)	(0.081)
$T_{i}\;\times$ 2nd year	0.018	−0.251	1.665*	0.097
	(0.032)	(0.691)	(0.937)	(0.073)
$T_{i}\;\times$ 3rd and beyond	−0.161	21.712***	−0.581	0.098
	(0.123)	(3.715)	(2.446)	(0.193)
				
Observations	1852	1852	1852	1852

(c) Differential effects by *easy/hard* (above/below median of first-year ECTS passed) degree-course and study-career year of first application				
*Dependent variables:*	Grad. on time	Time to grad.	Final mark	Distinction
	(1)	(2)	(3)	(4)
$T_{i}\;\times$ Above median – 1st year	0.081	−5.206***	5.777***	0.343**
	(0.087)	(1.558)	(1.958)	(0.139)
$T_{i}\;\times$ Above median – 2nd year	0.095	−3.097**	3.823**	0.048
	(0.078)	(1.574)	(1.483)	(0.102)
$T_{i}\;\times$ Above median – 3rd and beyond	0.136	16.004***	0.920	−0.188
	(0.109)	(4.034)	(3.462)	(0.233)
$T_{i}\;\times$ Below median – 1st year	0.025	−1.280	4.365***	0.192**
	(0.039)	(0.954)	(1.159)	(0.088)
$T_{i}\;\times$ Below median – 2nd year	−0.003	0.402	1.254	0.113
	(0.034)	(0.699)	(0.995)	(0.077)
$T_{i}\;\times$ Below median – 3rd and beyond	−0.371*	24.782***	−0.477	0.325
	(0.201)	(5.822)	(2.947)	(0.301)
				
Observations	1852	1852	1852	1852

*Notes:* The table reports the results of the estimation of IV regressions on the sample of bachelor’s students with a running variable within a bandwidth of 0.1, for four outcomes: the probability of graduating without delay; the time to graduation measured in months; the final graduation mark; a dummy for graduating with distinction. The reported coefficients are those of the interaction between given student characteristics of interest and the endogenous treatment variable, instrumented with the interactions of the predictions of the first-stage regression and the same characteristic. All specifications include a polynomial of the running variable of order 1 and are estimated using triangular kernels. Errors are clustered at the programme-year-specific ranking level. Robust standard errors are in parentheses.

We classify groups of degree courses (based on the International Standard Classification of Education (ISCED) 3-digit classification) as more or less demanding based on the median value of ECTS that students in those courses pass in their first year and investigate heterogeneity in the Erasmus effects across this dimension, interacted with the timing of application.^20^ Panel (c) of Table 4 shows the results. Overall, a larger positive effect on both graduation grade and (reduced) time to graduation is observed for students participating in mobility earlier, and especially for those enrolled in more demanding degree courses, i.e. in which students typically tend to accumulate fewer ECTS in the first year. This evidence is in favour of the hypothesis that the positive effects (or the absence of negative effects) of Erasmus participation on student academic performance might merely be a ‘mechanical result’ driven by students systematically obtaining higher grades for exams taken abroad. Anecdotal evidence suggests that teachers, both at home and at the host university, have more favourable attitudes towards Erasmus students, which may result in generous grading abroad and generous grade conversion at home whenever there is room for it (i.e. in the absence of the reference-group-specific conversion tables, as explained above).

To summarise the results of the heterogeneity analysis, we find that the positive effect on bachelor’s students’ final graduation marks is stronger and more significant for students in scientific and technical fields and students applying for – and potentially participating in – Erasmus earlier in their study career, for whom we also observe a reduction in the time to graduation. Both sources of heterogeneity feature aspects related to the type and content of exams potentially taken during the study period abroad. Moreover, the Erasmus ‘advantage’ is concentrated among students participating in mobility early and enrolled in more challenging courses, possibly indicating that higher grades during Erasmus allow some students to overcome first-year cream-skimming. Concerning master’s students, the majority (approximately 92%) apply for Erasmus in their first year of study. When exploring heterogeneity in the effects across master’s students from different fields of study and more/less demanding degree courses, the absence of effects from participating in the Erasmus programme for the different sub-groups is confirmed (see Table B7 in Appendix B).

In the next section, we exploit information on the specific characteristics of study abroad periods to further investigate the potential mechanisms behind the observed effects.

## 6 Potential mechanisms

In this section, we exploit information on specific attributes of the mobility programmes and investigate whether these play a role in determining the impact of Erasmus participation on academic performance, with the aim of shedding additional light on the potential channels through which the observed impact operates. In particular, we focus on the quality of the host institution and the length of the mobility programme. Both aspects shape the type of experience students have and likely influence what and how students learn and how they perform during and/or after the period abroad.

The relative quality of the host institution determines the nature of the change in learning inputs during the study period abroad, which in turn determines which impact is produced on learning outputs. On the one hand, a higher quality of teachers and peers and a greater quality and amount of resources available to students at the host institution, relative to the home university, may be thought of as positive inputs in the development of human capital. Thus, a student participating in a study abroad programme at an institution of relatively higher quality would accumulate more human capital than what she would have accumulated at her home university. This would entail that, all other factors being equal, the mobile student would perform better (both while abroad and after returning) relative to how she would have performed staying at home. On the other hand, a higher quality of teachers and peers might entail higher standards in terms of course curricula and learning assessments (e.g. the difficulty of course content and exams, stricter grading criteria) as well as a lower ability rank of the students from the home university relative to the students of the host institution. Thus, *ceteris paribus*, a student participating in the Erasmus programme may perform worse while abroad, relative to how she would have performed at home. This effect might be mitigated if teachers at host institutions have more generous attitudes towards visiting students from universities of relatively lower quality and are more lenient in grading. In this setting, the length of the study period abroad might play a role: any of the above-discussed mechanisms is potentially reinforced when students spend a longer period in more prestigious institutions abroad. A longer period of study abroad may imply a greater human capital gain, as well as the accumulation of a higher number of exams (with potentially lower grades) while abroad. Finally, additional behavioural mechanisms are potentially in place: mobile students might exert less effort while abroad, regardless of the destination, because they are mainly interested in aspects of ISM other than academic performance, which – in host universities of relatively better quality – would mitigate the positive impact on human capital and reinforce the negative impact on grades. On the other hand, students who apply for the Erasmus programme in host universities of relatively better quality might be more interested in their academic performance and may exert more effort while abroad, with implications in the opposite direction. All the above-mentioned channels should operate similarly but in opposite directions in the case of mobility towards host institutions of relatively lower quality.

We collect data on the quality of the host institutions and exploit the information on the duration of the mobility programme from University of Bologna administrative data to shed light on these mechanisms. While we are not able to investigate all the possible channels outlined above, we can speculate on the relative size of each component by looking at the overall results from the heterogeneity analysis across these characteristics.

The actual characteristics of the study abroad experience are only observed for students who are treated, and they are endogenous. Thus, we adapt the RDD in an attempt to estimate the causal impact of having participated in an Erasmus programme with a certain characteristic. More specifically, we construct indicators of the individual being at or above the cutoff score in *at least* one programme-specific ranking relative to applications to programmes with the relevant characteristics in her first year of application, and indicators of being at or above the cutoff score *only* in rankings relative to applications for programmes that do not have the characteristics of interest, again in her first year of application. We use these indicators to estimate reduced-form equations.

### Quality of the host institution.

The quality of higher education institutions is measured using information from the *Academic Ranking of World Universities* (ARWU) from the Shanghai Ranking Consultancy, which ranks approximately 2000 universities every year, based on several indicators of academic and research performance.^21^ The ARWU is constructed based on indicators of academic and research performance and, thus, proxies the overall quality of higher education institutions mainly by capturing aspects related to the quality of teachers and peers, which in turn may arguably be considered to be correlated with the amount and quality of resources offered to students. We use these data to identify higher quality universities, i.e. those among the top 100 ranked institutions. In our sample, these correspond to 14 universities located mainly (approximately 40%) in the United Kingdom, as well as in Germany, Belgium and the Netherlands, and northern European countries including Sweden, Denmark, Finland and Norway. These institutions are also of relatively higher quality with respect to the home university – the University of Bologna – which is among institutions ranked 201st to 300th (only institutions up to the 200th position are precisely ranked).

We regress the four main outcomes on two variables: one takes the value of 1 if the student is at or above the cutoff score in *at least* one ranking for applications to institutions of higher quality in her first year of application; the other takes the value of 1 if the student is at or above the cutoff score only in programme-specific rankings for applications to institutions of lower quality in her first year of application. Panel (a) of Table 5 reports the results of this empirical exercise.

Participating in mobility programmes in institutions of relatively lower quality has a positive and significant effect on the final graduation mark (column 3), while no significant impact of studying in foreign universities of relatively higher quality is found. In light of the conceptual framework presented above, these results might be interpreted in the following way: for Erasmus programmes in host institutions of relatively higher quality, if there is a positive impact on human capital, this is compensated by the negative impacts on performance while abroad deriving from the other channels. Instead, for Erasmus programmes in host institutions of relatively lower quality, if there is a negative impact on human capital, this is more than compensated by the positive impacts on performance, at least as measured by graduation grades (but not concerning time to graduation), from the other channels. Therefore, it seems that the overall effect is more strongly driven by factors affecting the grades obtained while abroad, rather than being directly related to the accumulation of human capital.

### Length of stay.

We further examine whether the length of time spent abroad plays a role in explaining the observed effects. We employ the same empirical approach described above, considering both the quality of the host institution and the length of the programme. The latter is measured by distinguishing longer study abroad periods of more than 6 months from shorter ones of up to 6 months. Results show no significant effect of study abroad in higher quality institutions, regardless of programme length (panel (b) of Table 5). However, positive effects on final graduation marks are observed for programs in lower quality institutions with longer durations. This evidence may suggest that for Erasmus programmes completed in lower quality host institutions, a potential negative impact on human capital accumulation might be offset by the potential positive effect on exam grades when staying longer, that is, taking a higher number of exams with better grades while abroad. This would confirm that the effect is primarily driven by factors affecting exam grades abroad and not related to increased human capital acquisition. All results presented in Table 5 remain unchanged when using a different definition of high-quality universities that captures host institution quality relative to the University of Bologna (35 universities ranked in the top 200; results are reported in Table B8).

**Table 5:** Heterogeneity of effects across programme characteristics – Bachelor sample – Reduced-form estimates.

(a) Differential effects by quality of host institution -- top 100				
*Dependent variables:*	Grad. on time	Time to grad.	Final mark	Distinction
	(1)	(2)	(3)	(4)
$Z_{ir}\;\times$ Top 100	0.045	−0.351	−0.214	−0.004
	(0.036)	(0.709)	(0.965)	(0.083)
$Z_{ir}\;\times$ Lower-ranked	0.008	−0.194	1.211**	0.065*
	(0.017)	(0.375)	(0.517)	(0.039)
				
Observations	1852	1852	1852	1852
R-squared	0.550	0.602	0.601	0.513

(b) Differential effects by quality of host institution -- top 100 -- and length of study abroad period				
*Dependent variables:*	Grad. on time	Time to grad.	Final mark	Distinction
	(1)	(2)	(3)	(4)
$Z_{ir}\;\times$ Top 100 – 6 months or more	0.054	0.341	−0.421	−0.067
	(0.055)	(1.050)	(1.191)	(0.096)
$Z_{ir}\;\times$ Lower ranked – 6 months or more	−0.001	−0.091	1.429***	0.052
	(0.019)	(0.419)	(0.547)	(0.040)
$Z_{ir}\;\times$ Top100 – less than 6 months	0.066	−1.982	−0.817	0.069
	(0.057)	(1.897)	(1.219)	(0.074)
$Z_{ir}\;\times$ Lower ranked – less than 6 months	0.025	−0.315	0.702	0.083
	(0.020)	(0.467)	(0.660)	(0.058)
				
Observations	1852	1852	1852	1852
R-squared	0.551	0.603	0.602	0.514

*** p < 0.01, ** p < 0.05, * p < 0.1

*Notes:* The table reports the results of the estimation of the reduced-form equation on the sample of bachelor’s students with a running variable within a bandwidth of 0.1, for four outcomes: the probability of graduating without delay; the time to graduation measured in months; the final graduation mark; and a dummy for graduating with distinction. The reported coefficients are those of the interactions between our instrument $Z_{ir}$ and the given characteristic of interest. All specifications include a polynomial of the running variable of order 1 and are estimated using triangular kernels. Errors are clustered at the programme-year-specific ranking level. Robust standard errors are in parentheses.

To shed more light on the mechanisms at play, we try to obtain additional evidence that can help disentangle the effect on performance while abroad from a more general effect on learning that would be reflected in performance after the study period abroad. We exploit data from an ad-hoc extraction of information on single exams for the sub-sample of students participating in the Erasmus programme from the administrative archives of the University of Bologna, including date of registration, number of ECTS, mark and a flag for exams taken abroad. From the initial sample of all applicants to Erasmus study abroad programmes between 2013/14 and 2018/19 who have graduated and for whom we have information on their study careers from the administrative data, we focus on bachelor’s students who participated in the Erasmus programme and build a panel dataset with student-exam-level data on exam grades. We construct indicators for exams taken before, during and after the study period abroad and look at the before-during/after Erasmus difference in average grades. Results are reported in Table B10, for the entire sample (column 1) and separately for students who spent a period abroad in a higher quality institution – as measured by both being a top-100-ranked university (column 2) and being ranked above the University of Bologna (column 4) – and in a lower quality institution (columns 3 and 5). The evidence suggests that the grades of Erasmus students tend to increase in the year of their period abroad but then go back to pre-Erasmus levels once they return to their home university, and this pattern is stronger for mobility in lower quality universities.

Overall, the evidence from the current section suggests that the benefits of studying abroad for bachelor’s students, in terms of academic performance, are closely tied to factors affecting student performance during the study abroad period and thus are less linked to human capital accumulation. Instead, the benefits are likely due to factors related to exam grades while abroad and are confined to the study abroad period. Table B9 in Appendix B again shows no significant impact of Erasmus participation on master’s students, even across different programme characteristics. Looking at the coefficient magnitudes, the results suggest that in contrast to bachelor’s students, outcomes are better for Erasmus students participating in programmes of shorter length, and even more so in higher quality universities. This might be consistent given that master’s students are closer to their entrance into the labour market and they may have different motivations for participating in Erasmus.

### Outcomes 1 year after graduation.

Most studies analysing the impact of study abroad programmes on labour market outcomes rely on survey data. Besides identification, a common issue is low response rates, which in previous literature has ranged between 25% (Parey & Waldinger, 2011) and 54% (Oosterbeek & Webbink, 2011). We attempt to provide more complete evidence by complementing the results on academic outcomes with evidence on early post-graduation outcomes. We match the administrative data from the University of Bologna used in the rest of the study with survey data collected by AlmaLaurea, an inter-university consortium that tracks post-graduation outcomes of graduates from the majority of Italian universities.^22^ Each year, AlmaLaurea administers online questionnaires to students who obtained a university degree one, three and five years prior. We were able to match the administrative data at our disposal with survey data collected one year after graduation. The response rate is remarkably high, ranging from 71% for the sample of master’s students to 77% for bachelor’s students. When comparing the samples of respondents to the initial samples across the pre-Erasmus individual characteristics used in our study, no significant differences emerge, except that foreign-born students are less represented in the master’s student respondents sample, possibly due to students going back to their country of origin after completing their university studies.

In Italy, the majority of students who complete a bachelor’s degree tend to continue their education with a master’s degree (75% overall in our baseline sample). For this reason, we consider a different set of outcomes for the two sub-samples of bachelor’s and master’s students. For the former, we construct indicators for being enrolled in a master’s programme, further looking at whether they are enrolled at the University of Bologna or abroad. For master students, we consider indicators of being enrolled in a PhD programme, being employed and working abroad.

**Table 6:** Erasmus participation and student outcomes one year after graduation.

(a) Bachelor’s students						
*Dependent variables:*	Enrolled in master		Master abroad		Master UniBo	
	(1)	(2)	(3)	(4)	(5)	(6)
	RF	IV	RF	IV	RF	IV
Above cutoff score	−0.003		0.051		−0.104	
	(0.044)		(0.038)		(0.065)	
Erasmus participation		−0.007		0.102		−0.208
		(0.088)		(0.076)		(0.129)
						
Observations	1271	1271	1271	1271	1271	1271
R-squared	0.564		0.433		0.425	

(b) Master’s students						
*Dependent variables:*	Enrolled in PhD		Employed		Employed abroad	
	(1)	(2)	(3)	(4)	(5)	(6)
	RF	IV	RF	IV	RF	IV
Above cutoff score	0.000		0.045		0.023	
	(0.042)		(0.074)		(0.102)	
Erasmus participation		0.000		0.077		0.042
		(0.073)		(0.129)		(0.189)
						
Observations	548	548	548	548	325	325
R-squared	0.548		0.533		0.513	

*** p < 0.01, ** p < 0.05, * p < 0.1

*Notes:* The table reports the results of the estimation of the reduced-form equation and the IV regression of Erasmus participation on student outcomes one year after graduation for the samples of bachelor’s students (panel (a)) and master’s students (panel (b)). Student administrative records are matched with follow-up survey data collected by the AlmaLaurea consortium in Italy. The outcomes are, in panel (a), an indicator for being enrolled in a master’s programme (columns 1 and 2), an indicator for enrolling in a master’s programme abroad (columns 3 and 4) and an indicator for enrolling in a master’s programme at the University of Bologna (columns 5 and 6); in panel (b), an indicator for enrolling in a PhD programme (columns 1 and 2), an indicator for being employed (columns 3 and 4) and an indicator for working abroad (columns 5 and 6). The estimations are performed on samples within a bandwidth of the running variable of 0.1. All specifications include a polynomial of the running variable of order 1 and are estimated using triangular kernels. Errors are clustered at the programme-year-specific ranking level. Robust standard errors are in parentheses.

We estimate both a reduced-form equation and an instrumental variable regression on the outcomes measured one year after graduation and report the results in Table 6. Given the reduction in sample size due to non-response to the follow-up surveys, the estimations performed on observations within a bandwidth around the cutoff are based on a relatively small sample. This implies that the resulting coefficients are not precisely estimated. Focusing on the magnitudes, the evidence from this analysis points to a zero overall effect of participating in Erasmus during undergraduate studies on enrolment in a master’s programme (columns 1 and 2 of panel (a)); nevertheless, it appears that Erasmus participation leads to bachelor’s students being more likely to continue education abroad and less likely to do it at the University of Bologna (columns 3–4 and 5–6 of panel (a) for the two outcomes, respectively). Concerning master’s students, we observe an overall zero effect on the probability of enrolling in a PhD programme (columns 1 and 2 of panel (b)), while the coefficients of the probability of being employed and – for those who are employed – the probability of working abroad are positive, yet not significant (columns 3–4 and 5–6 of panel (b) for the two outcomes, respectively).

Our findings for both sub-samples appear to suggest that the impact of Erasmus participation on student post-graduation paths is not mediated by an effect on academic performance. In our setting, on the one hand, the positive impact on undergraduate students’ final graduation marks does not appear to be reflected in an overall higher probability of continuing education. On the other hand, master’s students participating in Erasmus appear to have better labour market outcomes even though there is no noticeable effect on their academic performance. Thus, other factors, such as language competencies or soft skills like open-mindedness, may play a more significant role in determining a student’s career path after participating in the Erasmus programme, making them more mobile (based on the results obtained for outcomes such as enrolling in a master’s degree abroad for bachelor’s students and working abroad for master’s students). Yet, these findings are at best suggestive and are not conclusive.

## 7 Concluding remarks

This paper studies the effects of participating in the most popular European university study abroad programme – Erasmus – on student performance at graduation and one year after graduation. The results of our baseline analysis show no significant effect of spending a period of study abroad on time to graduation for either bachelor’s or master’s students and a positive effect on final graduation mark for bachelor’s students only. We then explore heterogeneous effects and find larger positive effects on the final graduation marks of bachelor’s students who graduate in a STEM field (science, technology, engineering and mathematics) and those who apply for the Erasmus programme in their first year of study, for whom we also observe a reduction in time to graduation. Both dimensions of heterogeneity are potentially related to the type and content of exams taken during the study abroad period, suggesting that these aspects might play a role in driving the observed impacts. Moreover, we find that the Erasmus ‘advantage’ is concentrated among students participating in mobility earlier and enrolled in more demanding degree courses (i.e. in which students typically accumulate fewer ECTS in the first year) – indicating that higher grades during Erasmus might allow some students to perform better in first-year high-stake exams and overcome first-year cream-skimming. Further investigating potential mechanisms, we find that the positive effect on final graduation marks for bachelor’s students is observed for students successfully applying for programmes in host institutions of (relatively) lower quality, in particular when the programme is also of longer duration. The quality of the receiving university and the length of the programme may play a role in influencing both student performance while abroad and, more broadly, what and how students learn. We provide additional descriptive evidence suggesting that the effect operates through a direct impact on student achievement during the study period abroad rather than a more general impact on learning performance, which would potentially be reflected in student achievement after the experience abroad. Finally, by merging administrative student records from the University of Bologna with survey data collected by the inter-university institution *AlmaLaurea*, we extend our analysis and investigate impacts on education and labour market outcomes one year after graduation, for which we do not find statistically significant effects.

Our research on the Erasmus programme provides valuable insight into the causal impact of study abroad experiences on university student performance. Given the growing popularity and size of study abroad programmes worldwide, understanding their effects is relevant to guide policy design and implementation. Additionally, the impacts on student academic performance are important both because spending a period studying abroad during one’s academic career produces a change in learning inputs and because student academic performance can affect outcomes following education.

The first key result of the paper indicates that participating in the Erasmus programme does not cause a delay in graduation, regardless of student and programme characteristics. This finding is relevant from a policy perspective and is the first robust evidence on a widely debated topic in the education and economics fields. It is above all crucial for the Italian context, where university studies tend to last longer compared to other European countries, leading to potential negative effects on students’ future outcomes. Our findings from the heterogeneity analysis may further suggest that students who are behind with their studies should be discouraged from participating in study abroad programmes, as these may lead to further delays in graduation.

The other main finding of the paper is that students who participate in the Erasmus programme complete their degrees with higher graduation grades. From a policy perspective, it is relevant to understand whether this result reflects human capital gains or other factors, and this is what the second part of the paper investigates. Overall, our analysis suggests that the benefits in terms of grade improvement are limited to student performance while abroad and are likely not driven by human capital gains. However, these findings are not conclusive, as despite the array of data used, we are not able to precisely measure all the potential mechanisms at play.

Finally, the results from our analysis of the impacts on education choices and labour market outcomes one year after graduation suggest that there might be a positive effect of participating in Erasmus on the probability of bachelor’s students continuing education abroad and on the probability of master’s students being employed; this effect is not necessarily mediated by impacts on academic performance, although sample size issues do not allow for precision in these estimates. Furthermore, it is worth reminding the reader that given our empirical design, we are only able to identify effects on the different outcomes for students with skill levels (including past academic performance and language proficiency) that allow them to ‘just’ qualify/not qualify for an Erasmus grant, whereas the effects are potentially different for students with a significantly higher/lower level of these skills.

We believe that further progress could be achieved by combining detailed data on study abroad programme applications and administrative data on labour market outcomes.^23^ This would allow for a more comprehensive analysis of the long-term effects of study abroad programmes, taking into consideration the potential mediating role of the impacts on academic performance.

## Declaration of competing interest

The authors declare that they have no known competing financial interests or personal relationships that could have appeared to influence the work reported in this paper.

## Data Availability

The authors do not have permission to share data.

## Appendix A Supplementary data

MMC S1 .

## Footnotes

1 Source: US Study Abroad data from the 2021 Open Doors report. Available at https://opendoorsdata.org/data/us-study-abroad/u-s-study-abroad-for-academic-credit-trends/.

2 The figures on the Erasmus programme reported in this section are from Eurostat, ‘Credit mobile graduates by education level, type of mobility scheme, type of mobility and gender’. In addition, we refer to https://ec.europa.eu/commission/presscorner/detail/en/MEMO_17_83.

3 Sources: ‘Erasmus Programme Guide 2023’, version 2, page 5 (https://erasmus-plus.ec.europa.eu/document/erasmus-programme-guide-2023-version-2-211222) and ‘Erasmus Programme Guide 2020’, version 3, page 5 (https://ec.europa.eu/programmes/erasmus-plus/sites/default/files/erasmus_programme_guide_2020_v3_en.pdf).

4 In European countries, academic credits are measured according to the European Credit Transfer System (ECTS). One ECTS credit corresponds to 25 to 30 hours of workload (lecture attendance or study hours).

5 Cullinan et al. (2022), Rodrigues (2013), Salisbury et al. (2013) and Waibel et al. (2018) use propensity score matching methods. Di Pietro (2015), Parey and Waldinger (2011) and Sorrenti (2017) rely on instrumental variable approaches, with supply-side measures of Erasmus grant availability used as an exogenous instrument for student mobility. One exception is Oosterbeek and Webbink (2011), which exploits data on applications to a small-scale Dutch study abroad programme targeted at a selective group of outstanding students and estimates the causal impact of the programme by means of a standard regression discontinuity design.

6 Data on enrolment in Italian universities are available at http://dati.ustat.miur.it/dataset/iscritti. Table B1 in Appendix B presents some statistics to compare the sample of students starting a study career at the University of Bologna with the overall population of Italian higher education students across some basic characteristics, including the share of female and foreign students and the distribution of students by field of study.

7 Source: AlmaLaurea institution, *Profilo dei Laureati 2019*https://www.almalaurea.it/universita/profilo/profilo2019. According to the same source, another 4.4% of 2019 graduates spent a period of study abroad through non-EU programmes: the University of Bologna manages another programme offering grants for mobility to non-EU countries (‘Overseas programme’), with similar selection criteria to the Erasmus+ programme; other ISM programmes not financed by the EU are also available but are mainly targeted at a few outstanding students or are focused on research (i.e. aimed at the preparation of the final thesis or targeted at Ph.D. students).

8 More specifically, for students enrolled at least to the second year of a bachelor’s, single-cycle or master’s degree, the calculation of study career points considers the student’s average grade normalised to the average grade in the department and cycle of the student’s degree course, and the number of ECTS credits registered during their study career with respect to the first year of enrolment (regularity of studies). For students enrolled in their first year of a master’s degree, 6/7 of the calculation of study career points comprises the previous courses of study (applicant’s graduation date and graduation mark normalised to the average grade in the department and cycle of the student’s degree course) and 1/7 relates to the individual student’s current course of study (the student’s average grade normalised to the average grade in the department and cycle of the student’s degree course, and the number of ECTS credits registered prior to the application).

9 The Italian higher education system is organised in two cycles: a 3-year first-level degree (the ‘Laurea Triennale’, equivalent to a bachelor’s degree) and a 2-year second-level degree (the ‘Laurea Specialistica/Magistrale’, equivalent to a master’s degree). Some specialised fields, such as medicine, dentistry, veterinary medicine, pharmacy, architecture, building engineering, law and primary education sciences, have degree programs organised into six- or five-year single cycles.

10 See Appendix A.1 for more details on how the rankings cutoff scores and the students’ running variables are derived, including a practical example.

11 For example, the average final grade among bachelor’s students is 98 for students below the threshold and outside the bandwidth, 104 for students within the bandwidth and 105 for students above the threshold and outside the bandwidth considered. The averages for master’s students are 105, 107 and 109, respectively.

12 We exclude applications for *Erasmus placement*, the programme offering scholarships to undertake traineeships abroad during one’s study career.

13 Administrative records on students enrolled in their first study career before the 2007/2008 academic year are not available. Approximately 6.4% of all applicants are not matched with administrative records on student study careers. These are either students who first enrolled before the 2007/2008 academic year or students who first enrolled in another university and transferred to the University of Bologna at a later stage in their study career.

14 Table B3 in Appendix B reports some summary statistics for the two groups not split by treatment status, as well as for the samples of bachelor’s and master’s students who never applied for Erasmus during their study career. The latter, on average, have a lower propensity to be mobile (as measured by the probability of having moved to enrol at the University of Bologna), are less likely to have graduated in social sciences fields and are more likely to have graduated in STEM and health and agricultural sciences fields. Their careers are also less successful (longer time to graduation and lower final graduation grade) relative to students who applied for Erasmus at least once in their career.

15 Only 0.9% of bachelor’s students and 0.4% of master’s students participated in an Erasmus programme twice over their study career.

16 The model in Eq. (2) does not provide a clear indication of the relationship between the running variable and the fitted values since the latter also depend on the estimated values of the fixed effects. For the graphical representation of the first stage, we estimate model (2) without fixed effects and, in order to try to reduce the bias in the estimated first-stage coefficient generated by the bulk of observations at the threshold point, we drop these observations.

17 In a setting with a high number of programme-year-specific rankings and ranking fixed effects, calculating the optimal bandwidth proposed by Calonico et al. (2014) turns out to be unfeasible because the calculation is too computationally intense. Our preferred bandwidth is arbitrarily chosen but guided by the Calonico–Cattaneo–Titiunik optimal bandwidths calculated for the different outcomes in a setting without ranking fixed effects (which range approximately between 0.11 and 0.14 in the sample of bachelor’s students.). Moreover, all estimations are performed using triangular kernels, which have optimal properties in estimating boundary points (see Imbens & Kalyanaraman, 2012).

18 We use the terms ‘exit grade’ and ‘(final) graduation mark/grade’ interchangeably in the current and following sections. Exam grades in Italy range between 18 and 30. The base graduation mark is obtained from the weighted average of grades for all exams, with the weights being the number of ECTS for each exam. Tables of statistical distributions of grades at the University of Bologna by field of study and academic year used to convert grades obtained in foreign institutions can be accessed at https://www.unibo.it/it/didattica/iscrizioni-trasferimenti-e-laurea/il-sistema-universitario/ects-label/tabelle-di-conversione-dei-voti-nella-scala-ects/tabelle-di-conversione-dei-voti-nella-scala-ects.

19 It is worth mentioning that a potential negative signalling effect of late graduation in the labour market might be mitigated if perceived as a consequence of the (potentially positively perceived) time investment in ISM. To the best of our knowledge, no evidence exists regarding this mechanism.

20 Degree courses in the arts, humanities and social sciences fields are mainly classified as ‘easy’ (less demanding) while degree courses in engineering, health and agricultural sciences fields are mainly classified as ‘hard’ (more demanding).

21 The yearly rankings from the Shanghai Ranking Consultancy are published online at http://www.shanghairanking.com. Details on the methodology can be found through the same link. We take the 2012 ranking – that of the year before the starting year of our sample – to avoid that the ranking is endogenously determined by the inflow of Erasmus students in foreign institutions.

22 Favero and Fucci (2017) and De Benedetto et al. (2023) also use AlmaLaurea data linked with individual university administrative data to investigate Erasmus impacts on labour market outcomes.

23 To date, there are only a few studies (Braga et al., 2016 and Bianchi, 2020) that have matched administrative data of students from specific Italian universities with tax records, thanks to special agreements between institutions.
