# Supplementary material for: Study abroad programmes and student outcomes: Evidence from Erasmus
Source: Econ Educ Rev. 2024 Apr;99:None. doi: 10.1016/j.econedurev.2024.102510 (PMC11008255; doi:10.1016/j.econedurev.2024.102510)
Supplement: MMC S1 — . [file mmc1.pdf]

A Appendix A

A.1 Design features

The selection process for the award of Erasmus grants presents several peculiarities that deserve further investigation. Within the main yearly call for Erasmus applications, launched in January of each year, the grant assignment mechanism can be summarized as follows:

- Each student can submit up to two applications for mobility grants funding specific Erasmus programmes.
- A student who is offered an Erasmus grant can decide to turn down the offer when the first ranking is published and within the deadline, and these grants are reallocated to the next students in the specific programme ranking, until the last eligible student.
- Within each ranking relative to a specific Erasmus programme, the qualifying cutoff score is the score of the last student who is offered one of the available grants, regardless of whether she has participated in Erasmus.
- The value of the running variable x for each student is the maximum of her scores – normalised to the cutoff score – among rankings in which she participates in the January call of her first year of application. The assignment indicator is $Z = \mathbf{1}(x \geq 0))$.

Given the outcome of this allocation:

- If there are non-assigned grants, a second round of applications is launched and the procedure is repeated. Students can only submit one application in this second round.
- After having initially accepted an Erasmus grant, students can later renounce to it or be rejected by the host institution due to not fulfilling specific requirements (e.g. deadlines, etc.). The grants of renouncing/rejected students are not reallocated.
- Any student can re-apply in subsequent years within her study career.

We define as treatment status (T_i) the binary indicator that takes value 1 if the student i has ever participated in the Erasmus programme during her study career.

Derivation of cutoff score and running variable Let us illustrate how the cutoff score and running variable are derived with an example. Consider that, in a given year t, in the main call for applications that takes place in January, $n = 15$ students are enrolled and two specific Erasmus programmes – programme A and programme B – exist to which they can apply. Let's assume that 5 students apply only for programme A, other 5 students apply only for programme B and the remaining 5 students apply for both. Let's further assume that students' application scores are as presented in Table A1.

Table A1: Example: students' application scores

Student	Score programme A	Score programme B
1	70	
2	85	
3	40	
4	60	
5	35	
6		68
7		80
8		50
9		45
10		75
11	75	85
12	36	55
13	50	40
14	90	70
15	80	78

Once students have applied, they are ranked based on the scores assigned to their applications. Then, those who are awarded an Erasmus grant decide whether to accept or turn down the grant offer. Let's assume that the outcome of this decision process aligns with the results presented in Table A2.

Table A2: Example: outcome of ranking and decision process

Ranking A	Score	Student decision	Norm. Score	Ranking B	Score	Student decision	Norm. Score
14	90	Turn down (Accept B)	0.500	11	85	**Accept**	0.214
2	85	**Accept**	0.417	7	80	Turn down	0.143
15	80	Turn down	0.333	15	78	Turn down	0.114
11	75	Turn down (Accept B)	0.250	10	75	Turn down	0.071
1	70	Turn down	0.167	14	70	**Accept**	0.000
4	60	**Accept**	0.000	6	68	No offer	-0.029
13	50	No offer	-0.167	12	55	No offer	-0.214
3	40	No offer	-0.333	8	50	No offer	-0.286
12	36	No offer	-0.400	9	45	No offer	-0.357
5	35	No offer	-0.417	13	40	No offer	-0.429

In our example, due to students turning down offers and the reallocation of turned-down grants, for both programmes the position in the ranking of the last student who is offered a grant (6th student for programme A and 5th student for programme B) is higher than the number of available grants (2 for both programmes).

Then, for each student, the *running variable* is computed as the maximum value of the normalized score. The results are presented in Table A3.

Table A3: Example: running variable and final *qualifying* ranking

Student	Running Variable	*Qualifying* Ranking
1	0.167	A
2	0.417	A
3	-0.333	A
4	0	A
5	-0.417	A
6	-0.029	B
7	0.143	B
8	-0.286	B
9	-0.357	B
10	0.071	B
11	0.25	A
12	-0.214	B
13	-0.167	A
14	0.5	A
15	0.333	A

Identification Our setting has two notable aspects: first, students offered a grant have the option to turn it down/renounce or be rejected later; second, those who were not offered the grant in their first year of application can reapply in subsequent years and, if successful, receive the grant. Thus, we have *two-sided non-compliance*. Identification in such designs may be invalidated by the presence of *defiers* (i.e. violation of the monotonicity assumption). As in standard settings with two-sided non-compliance, the absence of defiers cannot be directly tested. However, descriptive statistics can offer interesting insights.

Table A4 reports the share of students by treatment status (rows) and by the value of the running variable (i.e. a student's maximum normalised score among rankings to which she participated in her first year of application, the latter being indicated as t) being below or above the cutoff score (columns). Treatment status is distinguished across: non-treated students (i.e. students never participating in Erasmus during their study career) not applying for Erasmus in years subsequent to their first year of application (indicated as $t + 1$); non-treated students applying for Erasmus in subsequent years; treated students participating in Erasmus as the outcome of applying in t; treated students participating in Erasmus as the outcome of applying in $t + 1$. The results in Table A4 show that (i) the majority of students (approximately 80%) with running variables above the cutoff score, i.e. who are offered a mobility grant the first year of application, are treated at t, i.e. accept the scholarship the first time they have the chance of doing so; and (ii) the majority of those who are not offered the grant in t (below the cutoff) never participate in Erasmus (53.52 + 17.9 for bachelor's students and 66.37 + 16.37 for master's students, respectively. This evidence indicates that even if defiers existed, they would represent a small share of the sample.

The Erasmus grant assignment mechanism allows us to explicitly define theoretical sub-populations of interest and to define the sub-populations on which we identify our causal parameter of interest.

In the remainder of this section, for exemplification purposes we assume that a student cannot

Table A4: Descriptive statistics for investigating non-compliance

(a) Bachelor's students

**Treatment status**	**Treatment assignment in *t***		
	(1)	(2)	(3)
	Below cutoff	Above cutoff	Total
Not treated – Not applying in t+	972	273	1,245
	53.52%	13.02%	31.83%
Not treated – Applying in t+	325	31	356
	17.90%	1.48%	9.10%
Treated at t	0	2,291	2,291
	0.00%	82.53%	49.89%
Treated at t+	519	54	573
	28.58%	1.95%	12.48
Total	1,816	2,776	4,592
	100%	100%	100.00

(b) Master's students

**Treatment status**	**Treatment assignment in *t***		
	(1)	(2)	(3)
	Below cutoff	Above cutoff	Total
Not treated – Not applying in t+	631	219	850
	66.63%	15.11%	35.48%
Not treated – Applying in t+	155	15	170
	16.37%	1.04%	7.10%
Treated at t	0	1,707	1,707
	0.00%	79.40%	55.12%
Treated at t+	161	17	178
	17.00%	0.79%	5.75
Total	947	2,150	3,097
	100%	100%	100.00%

participate in the Erasmus programme more than once in her study career (this share is small in our data on the population of applicants, accounting for 1.65%). We also assume monotonicity (i.e. the absence of defiers).

First of all, we decompose our treatment indicator T_i as

$$T_i = T_i^t + T_i^{t+},$$

where T_i^t takes the value of 1 if student i participates in the Erasmus programme as an outcome of her first application year, while T_i^{t+} takes the value of 1 if student i participates in Erasmus as an outcome of applications in subsequent years. The assumption on the single participation implies that

$$T_i^{t+} = 0 \quad \text{if} \quad T_i^t = 1.$$

It is also important to underscore how $T_i^t = 1$ if and only if $Z_i^t = 1$ (i.e. *one-sided* non-compliance in t). Given the setup, we can define the following strata:

Table A5: Potential sub-populations

**Observed**				**Potential**				**Group**
Z_i^t	T_i	T_i^t	T_i^{t+}	$T_i^t(0)$	$T_i^{t+}(0)$	$T_i^t(1)$	$T_i^{t+}(1)$	
1	1	1	0	0	0	1	0	Complier I (CI)
				0	1			Always-Taker I (ATI)
1	1	0	1	0	0	0	1	Complier II (CII)
				0	1			Always-Taker II (ATII)
1	0	0	0	0	0	0	0	Never-Taker (NT)
0	0	0	0	0	0	1	0	Complier I (CI)
						0	1	Complier II (CII)
						0	0	Never Taker (NT)
0	1	0	1	0	1	1	0	Always-Taker I (ATI)
						0	1	Always-Taker II (ATII)

The two-period design implies that we may observe two types of 'always-taker' and two types of 'compliers', characterised by early (ATI, CI) or later (ATII, CII) participation.

Limits in T_i^t and T_i^{t+} identify combinations of sub-populations:

$$\lim_{x\to 0^+} \mathbb{E}[T^t|X = x] = \mathbb{P}\mathrm{r}[\mathrm{CI}|X = 0] + \mathbb{P}\mathrm{r}[\mathrm{ATI}|X = 0];$$
$$\lim_{x\to 0^-} \mathbb{E}[T^t|X = x] = 0;$$
$$\lim_{x\to 0^+} \mathbb{E}[T^{t+}|X = x] = \mathbb{P}\mathrm{r}[\mathrm{CII}|X = 0] + \mathbb{P}\mathrm{r}[\mathrm{ATII}|X = 0];$$
$$\lim_{x\to 0^-} \mathbb{E}[T^{t+}|X = x] = \mathbb{P}\mathrm{r}[\mathrm{ATI}|X = 0] + \mathbb{P}\mathrm{r}[\mathrm{ATII}|X = 0].$$

While the overall limits in T identify

$$\lim_{x\to 0^+} \mathbb{E}[T|X=x] = \mathbb{P}\mathrm{r}[\mathrm{CI}|X=0] + \mathbb{P}\mathrm{r}[\mathrm{ATI}|X=0] + \mathbb{P}\mathrm{r}[\mathrm{CII}|X=0] + \mathbb{P}\mathrm{r}[\mathrm{ATII}|X=0];$$
$$\lim_{x\to 0^-} \mathbb{E}[T|X=x] = \mathbb{P}\mathrm{r}[\mathrm{ATI}|X=0] + \mathbb{P}\mathrm{r}[\mathrm{ATII}|X=0].$$

Assuming continuity of the probability of belonging to a potential sub-population in a neighbourhood of the threshold point, the differences between the right and left limits (i.e. discontinuities) identify

$$\tau_{T^t} = \lim_{x\to 0^+} \mathbb{E}[T^t|X=x] - \lim_{x\to 0^-} \mathbb{E}[T^t|X=x] = \mathbb{P}\mathrm{r}[\mathrm{CI}|X=0] + \mathbb{P}\mathrm{r}[\mathrm{ATI}|X=0];$$
$$\tau_{T^{t+}} = \lim_{x\to 0^+} \mathbb{E}[T^{t+}|X=x] - \lim_{x\to 0^-} \mathbb{E}[T^{t+}|X=x] = \mathbb{P}\mathrm{r}[\mathrm{CII}|X=0] - \mathbb{P}\mathrm{r}[\mathrm{ATI}|X=0];$$
$$\tau_{T} = \lim_{x\to 0^+} \mathbb{E}[T|X=x] - \lim_{x\to 0^-} \mathbb{E}[T|X=x] = \mathbb{P}\mathrm{r}[\mathrm{CI}|X=0] + \mathbb{P}\mathrm{r}[\mathrm{CII}|X=0].$$

These quantities can be computed by estimating the 'first-stage' regressions for T, T^t and T^{t+} separately and by taking the values of the two boundary points:[24]

Table A6: Discontinuities in time-varying treatment indicators

	**Bachelor**			**Master**		
	T	T^t	T^{t+}	T	T^t	T^{t+}
	(1)	(2)	(3)	(4)	(5)	(6)
Above cutoff score	0.522***	0.849***	-0.327***	0.640***	0.819***	-0.179***
	(0.038)	(0.022)	(0.032)	(0.056)	(0.044)	(0.038)
Observations	1,512	1,512	1,512	760	760	760
R-squared	0.312	0.753	0.160	0.344	0.583	0.090
Left Lim	.363	0	.363	.204	0	.204
Right Lim	.885	.849	.036	.844	.819	.024

In principle, and assuming the absence of defiers, as in our setting, the 4 sub-populations (ATI, ATII, CI, CII) are not identifiable from the data: since the two boundaries in T are the sum of the four boundaries in T^t and T^{t+}, we can only rely on 3 pieces of information for 4 unknown quantities. However, columns (3) and (6) in the bottom row of Table A6 (reporting the $\lim_{x\to 0^+} \mathbb{E}[T^{t+}|X=x]$) show that the sum of CII and ATII is very small (3.6% for bachelor's students and 2.4% for master's students). Thus, we can reasonably argue that

$$\mathbb{P}\mathrm{r}[\mathrm{CII}|X=0] \simeq \mathbb{P}\mathrm{r}[\mathrm{ATII}|X=0] \simeq 0.$$

[24] To simplify the interpretation of the coefficients, we obtain discontinuities and boundary points by estimating a regression without ranking fixed effects and after having eliminated the mass of points at $x = 0$.

If the probability of being CII is *exactly* equal to zero and under standard continuity assumptions of the potential outcomes for each sub-population in a neighbourhood of the threshold point, the parameter obtained in our design identifies the treatment effect on the CI sub-population, i.e. students who participate in the Erasmus programme as the outcome of their first application year and would not have participated in Erasmus had they not been offered a grant. This sub-population represents roughly 50% of bachelor's applicants and more than 60% of master's applicants at the threshold point (columns 1 and 4 of table A6, respectively). On the other hand, the sub-populations of those who would still participate in Erasmus during their career had they not been offered a grant in their first application year (ATI) represent approximately more than 35% of bachelor's students and roughly 20% of master's students (columns 3 and 6 of table A6, respectively).

A.2 Similarities to centralised school assignment

Our research design shares some common features with the recent literature developing a methodology that generalises regression discontinuity designs to allow for multiple cutoffs and multiple running variables (Abdulkadiroglu et al., 2017, 2022). The context of this literature is centralised school assignment, where the matching of kids to schools is realised through a scheme that takes as input information on applicant preferences (parents provide a preference order of schools to which they apply) and school priorities (in each school, kids are assigned to priority groups based on observable family characteristics). Given preferences and priorities (labelled parental 'type'), the offer of scarce school seats is determined by tie-breaker rules that can be in the form of a lottery or are 'general' (non-lottery, e.g. test scores). Parental type is likely correlated with potential outcomes; general tie-breakers play the role of a running variable in a regression discontinuity design and are likewise a source of omitted variable bias. The authors show how, in their context, omitted variable bias is eliminated by controlling for a *local propensity score*, i.e. the ex-ante probability of receiving an offer quantified as a function of a few features of student type and tie-breakers such as proximity to the admissions cutoffs and the identity of key cutoffs for each applicant, which they show to have a much coarser distribution than the underlying type distribution. Conditional on the local propensity score, school assignments are shown to be asymptotically randomly assigned, and school seat assignment provides a credible instrument for school enrolment.

More specifically, for each school they classify applicants as *conditionally seated* if their (school-specific) tie-breaker value is in the neighbourhood of the school admission cutoff (i.e. in the range $[\tau - h; \tau + h]$, where τ is the admission cutoff and h is the selected bandwidth); *always seated* if the tie-breaker value is above the neighbourhood of the admission cutoff (higher than $(\tau + h)$); *never seated* if the tie-breaker value is below the neighbourhood of the admission cutoff (lower than $(\tau - h)$). The limiting local probability of assignment of a seat at each school is 0.5, 1 and 0, respectively, for the three groups of applicants. The probability of being assigned to a specific school is derived as the school seat assignment probability at that school multiplied by the probability of being excluded in preferred schools, i.e. the disqualification rates at preferred schools, which depend on priorities and key cutoffs at the preferred schools. The propensity score for assignment at any school with a given characteristic is then derived as the

sum of the propensity scores for the individual schools with the characteristic of interest (because the assignment algorithm generates a single offer for each child). The authors then estimate a two-stage least squares (2SLS) model with saturated controls for the local propensity scores and local linear controls for non-lottery tie-breakers. Saturated regression-conditioning on the local propensity score eliminates applicants with score values of zero or one, and only local linear control for general tie-breakers for applicants to schools in which students are 'conditionally seated' is implemented.

Relative to Abdulkadiroglu et al. (2022), our setting differs in terms of some key features. Namely, the matching of students to Erasmus grants does not depend on programme priorities (no applicant is granted priority in any programme); the preference order of applicants to more than one specific programme (i.e. the maximum of two programmes) is not explicit at the moment of application, and only when the first programme-specific rankings are published are preferences potentially revealed, at which time students sort into the preferred programme among those offered. Given only the participants' set of applications, ties are broken in favour of applicants with the highest tie-breaker value, i.e. application score, which is the single non-lottery tie-breaker characterising our setting. For these reasons, the propensity score in our setting is simply the probability of being conditionally seated in *at least* one programme. This can take only the values of 0, $1/2$, $3/4$ or 1. In more detail, the propensity score takes a value of 0 for students who submit one application and are classified as never seated and students who submit two applications and are classified as never seated in both; 1 for students who submit one application and are classified as always seated and students who submit two applications and are classified as always seated in at least one application; $1/2$ for students who submit one application and are conditionally seated and students who submit two applications and are conditionally seated in one and never seated in the other; $3/4$ for students who submit two applications and are conditionally seated in both. Estimating our model by means of local linear regressions makes our approach comparable to that proposed by Abdulkadiroglu et al. (2022). In particular, excluding individuals with a maximum score above (below) the selected bandwidth, i.e. the always (never) seated, implies excluding individuals with a propensity score equal to 1 (0). Including controls for the remaining two values of the propensity score leaves the results unchanged, as shown in Table A7. The table reports the results from the estimation of the model described in equations 1 and 2 with the inclusion of indicators of the propensity score taking the values $1/2$ or $3/4$, for the four main outcomes.

Table A7: Main results controlling for propensity score

(a) Bachelor sample

Dependent variables:	Prob. of graduating on time		Time to grad. (months)		Final graduation mark		Prob. of distinction	
	(1)	(2)	(3)	(4)	(5)	(6)	(7)	(8)
	RF	IV	RF	IV	RF	IV	RF	IV
Above cutoff score	0.011		-0.202		1.091**		0.059	
	(0.017)		(0.369)		(0.510)		(0.038)	
Erasmus participation		0.021		-0.387		2.096**		0.114
		(0.032)		(0.709)		(0.956)		(0.073)
Observations	1,852	1,852	1,852	1,852	1,852	1,852	1,852	1,852
R-squared	0.550		0.603		0.601		0.513	

*** p<0.01, ** p<0.05, * p<0.1

(b) Master sample

Dependent variables:	Prob. of graduating on time		Time to grad. (months)		Final graduation mark		Prob. of distinction	
	(1)	(2)	(3)	(4)	(5)	(6)	(7)	(8)
	RF	IV	RF	IV	RF	IV	RF	IV
Above cutoff score	0.002		-0.014		0.706		0.043	
	(0.042)		(0.588)		(0.653)		(0.075)	
Erasmus participation		0.004		-0.023		1.167		0.071
		(0.069)		(0.971)		(1.088)		(0.124)
Observations	899	899	899	899	899	899	899	899
R-squared	0.545		0.582		0.488		0.499	

*** p<0.01, ** p<0.05, * p<0.1

Notes: The table reports the results of the estimation of a reduced-form equation (columns 1, 3, 5 and 7 of both panels) and of an IV regression (columns 2, 4, 6 and 8 of each panel) including the control for whether the propensity score takes a value of 1/2 or 3/4, for samples of bachelor's – panel (a) – and master's – panel (b) – students with a running variable within a bandwidth of 0.1, for the four outcomes of interest. All specifications include a polynomial of the running variable of order 1 and are estimated using triangular kernels. Errors are clustered at the programme-year-specific ranking level. Robust standard errors are in parentheses.

B Appendix B

B.1 Additional Figures and Tables

Figure B1: Density of the running variable

(a) Sample of bachelor's students

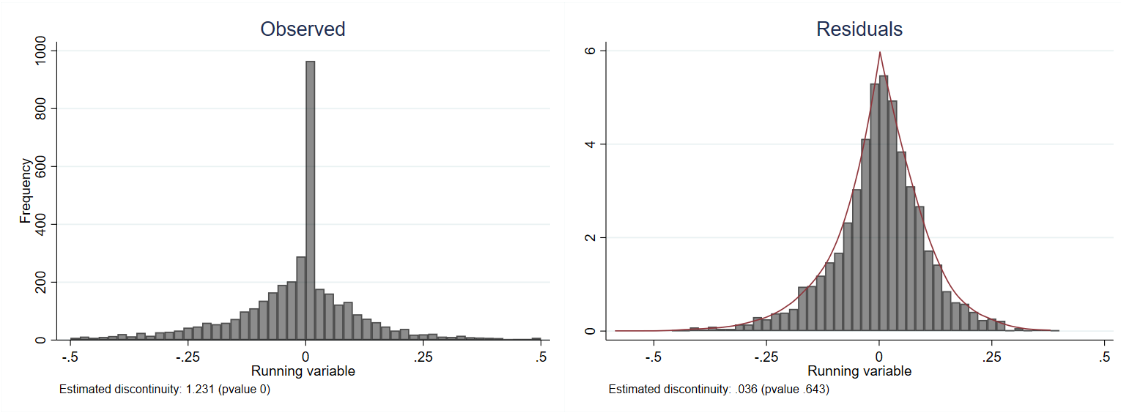

(b) Sample of master's students

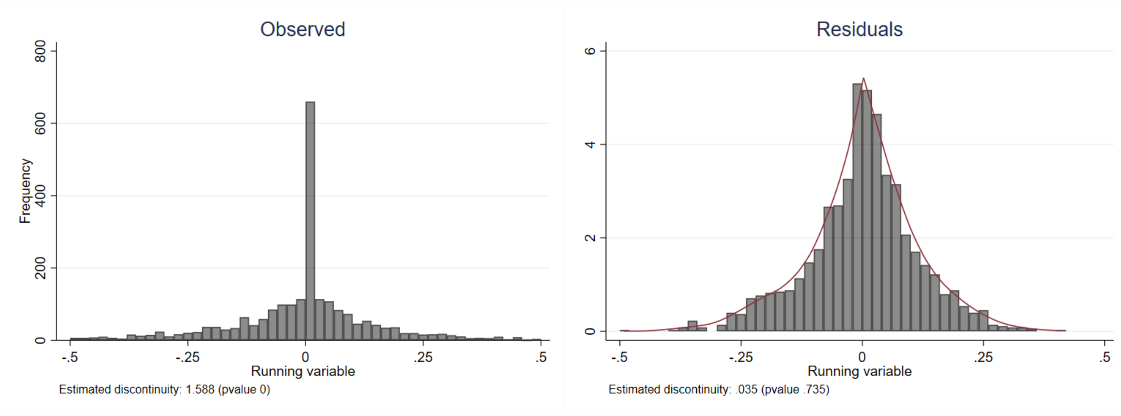

Notes: The figure plots the density of the running variable within a distance of 0.5 from the cutoff normalised to 0. The left-hand graph of both panels displays the unadjusted density. The right-hand graphs plot the density of the running variable net of the programme-year-specific ranking fixed effects.

Figure B2: Intention-to-treat graphs

(a) Sample of bachelor's students

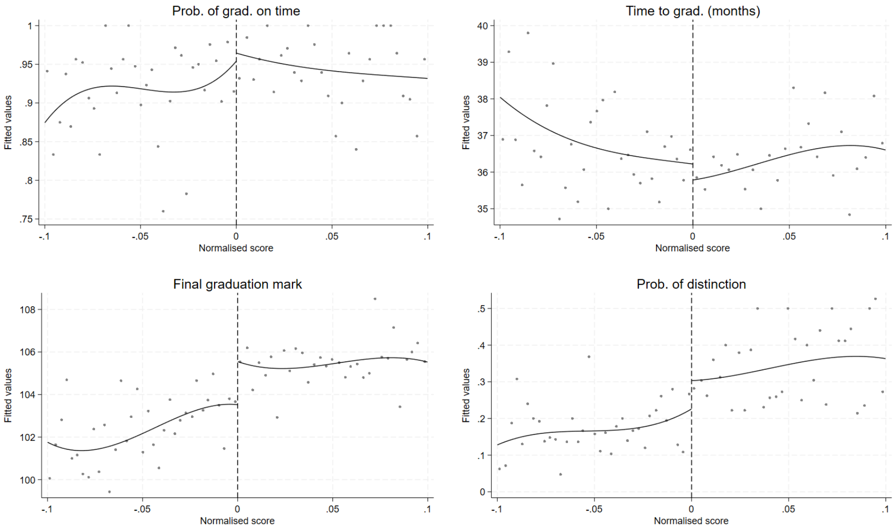

(b) Sample of master's students

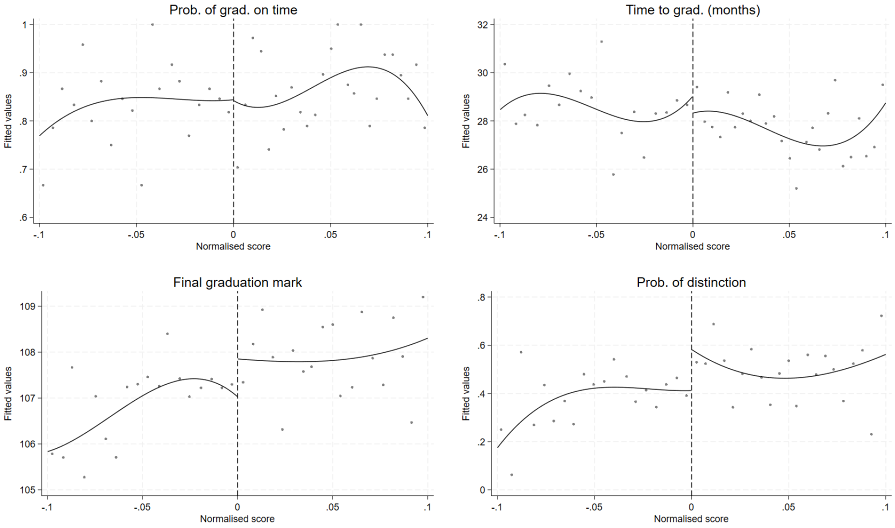

Notes: The four figures of panel (a) for the sample of bachelor's students and of panel (b) for the sample of master's students plot averages within bins of the running variable on the x-axis and the four main outcomes of interest on the y-axis, respectively: probability of graduating on time, time to graduation, final graduation mark and probability of distinction. The running variable and the outcome variables are defined in Section 3 and Section 4, respectively. The number of bins is calculated with the mimicking variance evenly spaced method using spacings estimators. The relationship is fitted with a polynomial of order three.

Figure B3: Main results: IV estimates with different bandwidths

(a) Bachelor

Prob. of graduating on time | Time to grad. (months) | Final mark | Prob. of distinction

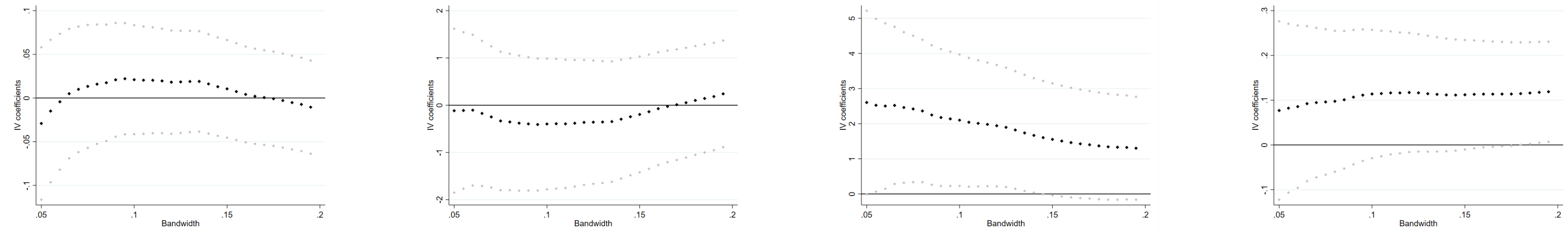

(b) Master

Prob. of graduating on time | Time to grad. (months) | Final mark | Prob. of distinction

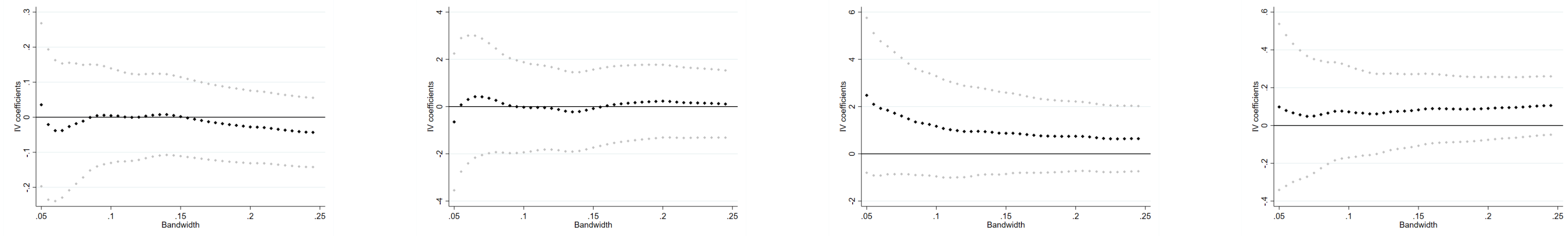

Notes: The four figures of each panel plot the coefficients (dark dotted line) and the 95% confidence intervals (grey dotted lines) from the estimation of an IV regression with a first-order polynomial of the running variable and a triangular kernel as a function of different bandwidths for the four main outcomes, for the samples of bachelor's – panel (a) – and master's – panel (b) – students, respectively. The four outcomes are an indicator of the probability of graduating without delay, the number of months to graduation, the final graduation mark (which ranges between 66 and 110) and a dummy for graduating with distinction.

Table B1: Representativeness of the sample of students from the University of Bologna

	**University of Bologna**	**All Italian universities**
	(1)	(2)
Statistics:		
No. of students first enrolled	136,046	2,848,842
Share of female students	0.56	0.55
Share of foreign students	0.08	0.05
Share of students by field of study:		
Education-Arts-Humanities	0.25	0.19
Social sciences	0.15	0.15
Business-Admin-Law	0.17	0.19
STEM	0.27	0.3
Health-Welfare	0.11	0.13
Agriculture-Veterinary	0.04	0.03

Notes: The table reports some selected statistics on the composition of the sample of students enrolled in their first university career at the University of Bologna from the 2010/2011 to 2019/2020 academic year – in column 1 – and the population of higher education students first enrolled in any Italian university in the same time period – column 2. Source: Italian Ministry of Education (data extracted from http://dati.ustat.miur.it/dataset/immatricolati).

Table B2: Descriptives on the initial sample of applications

	Mean	(s.d.)
	(1)	(2)
**Variables:**		
Academic year:		
2013/2014	0.14	(0.35)
2014/2015	0.15	(0.36)
2015/2016	0.17	(0.37)
2016/2017	0.18	(0.38)
2017/2018/	0.18	(0.38)
2018/2019	0.18	(0.38)
No. of grants per call	2.31	(1.8)
No. of applications per call	3.6	(4.17)
Length:		
3-4 months	0.03	(0.17)
5-6 months	0.56	(0.5)
7-8 months	0	(0.07)
9-10 months	0.37	(0.48)
11-12 months	0.03	(0.17)
Country of destination:		
Spain	0.25	(0.43)
France	0.15	(0.36)
Germany	0.11	(0.31)
UK	0.06	(0.24)
Belgium	0.06	(0.24)
Portugal	0.05	(0.22)
Others	0.32	(0.47)
Observations	10,127	

Notes: The table displays summary statistics for the sample of all applications for an Erasmus grant funding a period of study abroad submitted by students from the University of Bologna between the 2013/2014 and 2018/2019 academic years.

Table B3: Descriptive statistics on the final sample of Erasmus applicants and non-Erasmus applicants

	Degree Level							
	Bachelor				Master			
**Variables:**	**Mean**	**S.d.**	**Mean**	**S.d.**	**Mean**	**S.d.**	**Mean**	**S.d.**
	No Erasmus application		Erasmus applicants		No Erasmus application		Erasmus applicants	
**Student characteristics**								
Female	0.57	(0.49)	0.61	(0.49)	0.57	(0.49)	0.53	(0.5)
Moved from other region	0.36	(0.48)	0.54	(0.5)	0.52	(0.5)	0.65	(0.48)
Foreign-born	0.07	(0.25)	0.06	(0.23)	0.08	(0.27)	0.12	(0.32)
Field of study:								
Education-Arts-Humanities	0.26	(0.44)	0.3	(0.46)	0.27	(0.44)	0.2	(0.4)
Social sciences	0.18	(0.39)	0.4	(0.49)	0.22	(0.41)	0.33	(0.47)
Business-Admin-Law	0.11	(0.32)	0.14	(0.35)	0.11	(0.31)	0.17	(0.38)
STEM	0.28	(0.45)	0.11	(0.31)	0.34	(0.47)	0.27	(0.45)
Health & Agricultural sciences	0.16	(0.37)	0.06	(0.23)	0.07	(0.25)	0.03	(0.17)
**Applications**								
Total no. of applications:	-	-	2.09	(0.98)	-	-	1.85	(0.71)
No. of applications by ac.year:	-	-	1.79	(0.61)	-	-	1.78	(0.61)
Career year of first application (bachelor):								
First	-	-	0.31	(0.46)	-	-	-	-
Second	-	-	0.62	(0.49)	-	-	-	-
Third and beyond	-	-	0.08	(0.26)	-	-	-	-
Career year of first application (master):								
First (or 3rd bachelor)	-	-	-	-	-	-	0.94	(0.23)
Second and beyond	-	-	-	-	-	-	0.06	(0.23)
Academic performance at application:								
No. of exams at 1st application	-	-	4.99	(3.82)	-	-	0.61	(1.43)
No. of ECTS at 1st application	-	-	43.22	(31.95)	-	-	4.6	(10.66)
**Outcomes at graduation**								
Graduated on time	0.69	(0.46)	0.89	(0.31)	0.69	(0.46)	0.8	(0.4)
Time to graduation (months)	43.8	(12.65)	37.52	(6.98)	31.44	(9.58)	29.06	(5.95)
Final graduation grade	99.29	(8.38)	102.69	(7.31)	106.43	(5.27)	107.07	(4.53)
Prob. of distinction	0.13	(0.34)	0.21	(0.41)	0.41	(0.49)	0.43	(0.49)
Observations	57,923		3,912		43,982		2,396	

Notes: The table reports summary statistics for the final samples of students who never applied for Erasmus and students who applied for Erasmus at least once in their study career, separately by degree level (bachelor and master). The final samples are made up of students who enrolled in the first year of a study career at the University of Bologna from the 2007/2008 academic year onwards and who had graduated by the end of 2019 (when the data were extracted).

Table B4: First stage

	(a) Bachelor sample	(b) Master sample
Dependent variable:	Erasmus participation	
	(1)	(2)
Above cutoff score	0.521***	0.608***
	(0.039)	(0.058)
Observations	1,852	899
R-squared	0.627	0.695
Bandwidth	.1	.1
Model	Linear	Linear
Kernel:	Triangular	Triangular

*** p<0.01, ** p<0.05, * p<0.1

Notes: The table reports the results from the estimation of the first-stage equation (eq. 2) for the samples of bachelor's (column 1) and master's (column 2) students, including a polynomial of the running variables of order 1, estimated using triangular kernels. Errors are clustered at the programme-year-specific ranking level. Robust standard errors are in parentheses.

Table B5: Alternative measures of time to graduation

(a) Bachelor sample

Dependent variables:	Grad. by July		Grad. by October		Grad. by December		Grad. by March	
	(1)	(2)	(3)	(4)	(5)	(6)	(7)	(8)
	RF	IV	RF	IV	RF	IV	RF	IV
Above cutoff score	0.028		-0.011		-0.018		0.014	
	(0.042)		(0.027)		(0.028)		(0.019)	
Erasmus participation		0.053		-0.021		-0.035		0.028
		(0.082)		(0.052)		(0.055)		(0.037)
Observations	1,852	1,852	1,852	1,852	1,852	1,852	1,852	1,852
R-squared	0.529		0.716		0.561		0.543	

*** p<0.01, ** p<0.05, * p<0.1

(b) Master sample

Dependent variables:	Grad. by July		Grad. by October		Grad. by December		Grad. by March	
	(1)	(2)	(3)	(4)	(5)	(6)	(7)	(8)
	RF	IV	RF	IV	RF	IV	RF	IV
Above cutoff score	-0.040		-0.079		-0.037		0.014	
	(0.039)		(0.059)		(0.069)		(0.041)	
Erasmus participation		-0.066		-0.131		-0.061		0.022
		(0.064)		(0.100)		(0.114)		(0.068)
Observations	899	899	899	899	899	899	899	899
R-squared	0.396		0.587		0.588		0.543	

*** p<0.01, ** p<0.05, * p<0.1

Notes: The table reports the results of the estimation of a reduced-form equation (columns 1, 3, 5 and 7 of both panels) and of an IV regression (columns 2, 4, 6 and 8 of each panel) for four alternative measures of time to graduation: the probability of graduating in July of the last year of the degree's legal duration (3rd year for bachelor's students and 2nd for master's students), in columns 1 and 2 of both panels; October of the last year of the degree's legal duration, in columns 3 and 4 of both panels; December of the last year of the degree's legal duration, in columns 5 and 6 of both panels; March of the year following the last year of the degree's legal duration, in columns 7 and 8 of both panels. The estimations are performed on samples within a bandwidth of 0.1. All specifications include a polynomial of the running variable of order 1 and are estimated using triangular kernels. Errors are clustered at the programme-year-specific ranking level. Robust standard errors are in parentheses.

Table B6: Effect on GPA before graduation

	(a) Bachelor sample		(b) Master sample	
Dependent variable:	GPA before graduation			
	(1)	(2)	(3)	(4)
	RF	IV	RF	IV
Above cutoff score	0.278**		0.079	
	(0.132)		(0.188)	
Erasmus participation		0.534**		0.130
		(0.248)		(0.309)
Observations	1,852	1,852	899	899
R-squared	0.625		0.546	

*** p<0.01, ** p<0.05, * p<0.1

Notes: The table reports the results of the estimation of the reduced-form equation (columns 1 and 3 for the samples of bachelor's and master's students, respectively) and of the IV regression (columns 2 and 4 for the samples of bachelor's and master's students, respectively) for the average exam mark at the end of one's study career, before graduation. The estimations are performed on samples within a bandwidth of 0.1. All specifications include a polynomial of the running variable of order 1 and are estimated using triangular kernels. Errors are clustered at the programme-year-specific ranking level. Robust standard errors are in parentheses.

Table B7: Heterogeneity of effects across students characteristics – Master sample – IV estimates

(a) Differential effects by field of study

Dependent variables:	Grad. on time	Time to grad.	Final mark	Distinction
	(1)	(2)	(3)	(4)
$T_{ir}\times$ Arts & Humanities	0.061	-1.220	0.480	0.269
	(0.132)	(1.878)	(1.035)	(0.176)
$T_{ir}\times$ Social sciences, business & law	0.006	0.128	1.126	0.039
	(0.075)	(1.009)	(1.157)	(0.132)
$T_{ir}\times$ Science, engineering & maths	-0.088	1.057	1.736	-0.014
	(0.124)	(1.335)	(1.823)	(0.189)
$T_{ir}\times$ Health & agricultural sciences	0.428	-8.493**	5.546***	0.513
	(0.303)	(4.248)	(1.739)	(0.368)
Observations	899	899	899	899

(b) Differential effects by *easy/hard* (above/below median) degree course

Dependent variables:	Grad. on time	Time to grad.	Final mark	Distinction
	(1)	(2)	(3)	(4)
$T_{ir}\times$ Above median	-0.057	0.318	0.854	0.031
	(0.080)	(1.127)	(1.127)	(0.136)
$T_{ir}\times$ Below median	0.060	-0.340	1.448	0.109
	(0.075)	(1.023)	(1.226)	(0.135)
Observations	899	899	899	899

Notes: The table reports the results of the estimation of IV regressions on the sample of master's students with a running variable within a bandwidth of 0.1, for four outcomes: the probability of graduating without delay; the time to graduation measured in months; the final graduation mark; and a dummy for graduating with distinction. The reported coefficients are those of the interaction between given student characteristics of interest and the endogenous treatment variable instrumented with the interactions of the predictions of the first-stage regression and the same characteristic. All specifications include a polynomial of the running variable of order 1 and are estimated using triangular kernels. Errors are clustered at the programme-year-specific ranking level. Robust standard errors are in parentheses.

Table B8: Heterogeneity of effects across programme characteristics – Bachelor sample – Reduced-form estimates. Quality measured relative to the University of Bologna.

(a) Differential effects by quality of host institution – above University of Bologna

Dependent variables:	Grad. on time	Time to grad.	Final mark	Distinction
	(1)	(2)	(3)	(4)
Ranked above UniBo	0.029	0.078	0.780	0.078
	(0.024)	(0.483)	(0.692)	(0.065)
Ranked below UniBo	0.006	-0.284	1.179**	0.054
	(0.017)	(0.387)	(0.531)	(0.040)
Observations	1,852	1,852	1,852	1,852
R-squared	0.550	0.602	0.601	0.513

*** p<0.01, ** p<0.05, * p<0.1

(b) Differential effects by quality of host institution – above University of Bologna – and length of study abroad period

Dependent variables:	Grad. on time	Time to grad.	Final mark	Distinction
	(1)	(2)	(3)	(4)
Above UniBo – 6 months or more	0.013	0.330	0.954	0.068
	(0.023)	(0.521)	(0.718)	(0.069)
Below UniBo – 6 months or more	0.001	-0.253	1.433**	0.040
	(0.021)	(0.459)	(0.590)	(0.041)
Above UniBo – below 6 months	0.110*	-1.272	0.077	0.124
	(0.064)	(1.155)	(1.162)	(0.115)
Below UniBo – below 6 months	0.013	-0.293	0.725	0.079
	(0.018)	(0.487)	(0.684)	(0.059)
Observations	1,852	1,852	1,852	1,852
R-squared	0.552	0.603	0.601	0.513

*** p<0.01, ** p<0.05, * p<0.1

Notes: The table reports the results of the estimation of the reduced-form equation on the sample of bachelor's students with a running variable within a bandwidth of 0.1, for four outcomes: the probability of graduating without delay; the time to graduation measured in months; the final graduation mark; and a dummy for graduating with distinction. All specifications include a polynomial of the running variable of order 1 and are estimated using triangular kernels. Errors are clustered at the programme-year-specific ranking level. Robust standard errors are in parentheses.

Table B9: Heterogeneity of effects across programme's characteristics – Master sample – Reduced-form estimates

(a) Differential effects by quality of host institution – top 100

Dependent variables:	Grad. on time (1)	Time to grad. (2)	Final mark (3)	Distinction (4)
$Z_{ir}\times$ Top 100	0.016	-0.476	1.320	0.042
	(0.078)	(1.261)	(1.060)	(0.127)
$Z_{ir}\times$ Lower-ranked	0.000	0.063	0.603	0.044
	(0.042)	(0.586)	(0.678)	(0.077)
Observations	899	899	899	899
R-squared	0.544	0.582	0.488	0.499

(b) Differential effects by quality of host institution – top 100 – and length of study abroad period

Dependent variables:	Grad. on time (1)	Time to grad. (2)	Final mark (3)	Distinction (4)
$Z_{ir}\times$ Top 100 – 6 months or more	0.079	-1.942*	0.200	-0.012
	(0.058)	(1.173)	(1.022)	(0.169)
$Z_{ir}\times$ Lower ranked – 6 months or more	-0.010	-0.082	0.427	0.099
	(0.046)	(0.671)	(0.717)	(0.075)
Top100 – below 6 months	0.223	-3.359	3.216	0.303
	(0.238)	(2.117)	(2.056)	(0.240)
Lower ranked – below 6 months	0.064	-0.270	0.320	-0.081
	(0.049)	(0.691)	(0.915)	(0.099)
Observations	899	899	899	899
R-squared	0.547	0.586	0.489	0.505

Notes: The table reports the results of the estimation of the reduced-form equation on the sample of master's students with a running variable within a bandwidth of 0.1, for four outcomes: the probability of graduating without delay; the time to graduation measured in months; the final graduation mark; and a dummy for graduating with distinction. The reported coefficients are those of the interactions between our instrument Z_{ir} and the given characteristic of interest. All specifications include a polynomial of the running variable of order 1 and are estimated using triangular kernels. Errors are clustered at the programme-year-specific ranking level. Robust standard errors are in parentheses.

Table B10: Evidence from data on exam grades

Dependent variable:	GPA				
	(1)	(2)	(3)	(4)	(5)
	All students	Top 100	Lower ranked	Above UniBO	Below UniBo
During Erasmus	0.64***	0.47*	0.65***	-0.04	0.82***
	(0.17)	(0.24)	(0.17)	(0.12)	(0.19)
After Erasmus	0.01	-0.03	0.01	-0.31**	0.10
	(0.07)	(0.21)	(0.08)	(0.15)	(0.09)
Constant	26.97***	27.58***	26.92***	27.75***	26.76***
	(0.04)	(0.07)	(0.04)	(0.04)	(0.04)
Observations	79,490	5,987	73,503	15,919	63,571
R-squared	0.07	0.11	0.07	0.08	0.07
Career-year FE	Yes	Yes	Yes	Yes	Yes
Degree-course FE	Yes	Yes	Yes	Yes	Yes

*** p<0.01, ** p<0.05, * p<0.1

Notes: The sample is composed of all applicants to Erasmus study abroad programmes between 2013/14 and 2018/19 who have graduated from a bachelor's degree and for whom there is information in the administrative data on study careers, and who participated in the Erasmus programme. Observations are at the student-exam level. 'During Erasmus' is defined as the calendar year of exam dates registered as taken while abroad, and 'after Erasmus' identifies subsequent calendar years. Errors are clustered at the degree-course level. Robust standard errors are in parentheses.
